# Microarray Strategies for Exploring Bacterial Surface Glycans and Their Interactions With Glycan-Binding Proteins

**DOI:** 10.3389/fmicb.2019.02909

**Published:** 2020-01-10

**Authors:** María Asunción Campanero-Rhodes, Angelina Sa Palma, Margarita Menéndez, Dolores Solís

**Affiliations:** ^1^Instituto de Química Física Rocasolano, Consejo Superior de Investigaciones Científicas, Madrid, Spain; ^2^Centro de Investigación Biomédica en Red de Enfermedades Respiratorias, Instituto de Salud Carlos III, Madrid, Spain; ^3^UCIBIO, Department of Chemistry, Faculty of Science and Technology, NOVA University of Lisbon, Lisbon, Portugal

**Keywords:** bacterial glycans, microarrays, lectins, antibodies, immune system, vaccine development, bacterial interactions

## Abstract

Bacterial surfaces are decorated with distinct carbohydrate structures that may substantially differ among species and strains. These structures can be recognized by a variety of glycan-binding proteins, playing an important role in the bacteria cross-talk with the host and invading bacteriophages, and also in the formation of bacterial microcolonies and biofilms. In recent years, different microarray approaches for exploring bacterial surface glycans and their recognition by proteins have been developed. A main advantage of the microarray format is the inherent miniaturization of the method, which allows sensitive and high-throughput analyses with very small amounts of sample. Antibody and lectin microarrays have been used for examining bacterial glycosignatures, enabling bacteria identification and differentiation among strains. In addition, microarrays incorporating bacterial carbohydrate structures have served to evaluate their recognition by diverse host/phage/bacterial glycan-binding proteins, such as lectins, effectors of the immune system, or bacterial and phagic cell wall lysins, and to identify antigenic determinants for vaccine development. The list of samples printed in the arrays includes polysaccharides, lipopoly/lipooligosaccharides, (lipo)teichoic acids, and peptidoglycans, as well as sequence-defined oligosaccharide fragments. Moreover, microarrays of cell wall fragments and entire bacterial cells have been developed, which also allow to study bacterial glycosylation patterns. In this review, examples of the different microarray platforms and applications are presented with a view to give the current state-of-the-art and future prospects in this field.

## Introduction

**FIGURE 1:**
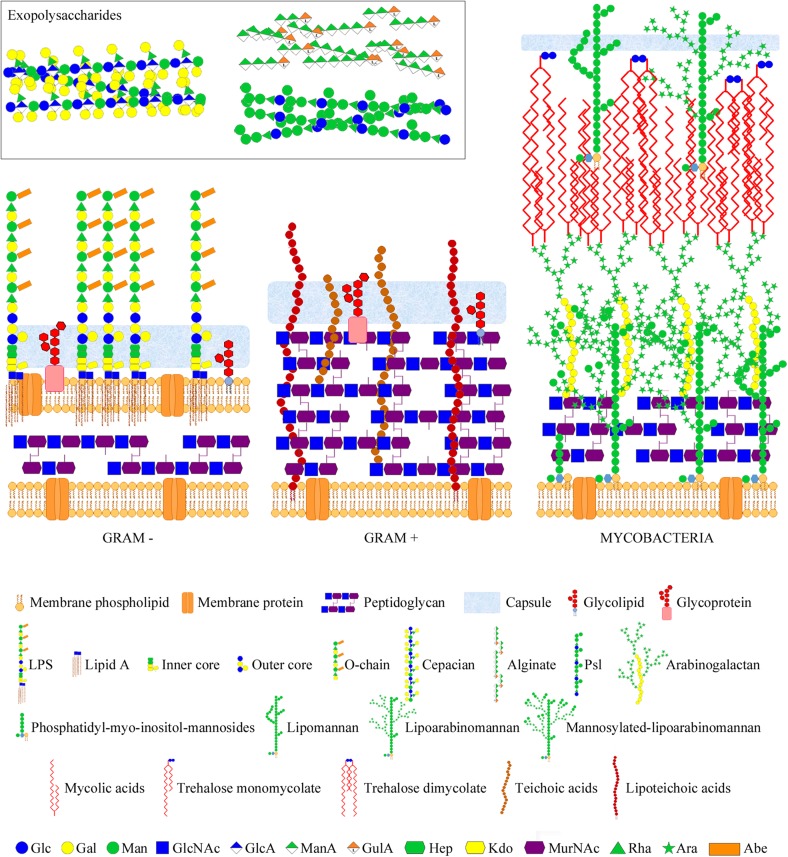
Bacterial glycans and architecture of the cell wall of different bacterial groups. Gram-negative bacteria **(left part)** contain a thin peptidoglycan layer, sandwiched between two cell membranes, and display LPSs (composed of lipid A, inner and outer core, and O-chain) anchored to the outer membrane. Gram-positive bacteria **(middle part)** contain a thick peptidoglycan layer, covering the cell membrane, and usually display teichoic acids anchored to the membrane (lipoteichoic acids) or bound to the peptidoglycan. Gram-negative and -positive bacteria may also present cell surface glycolipids, glycoproteins, and a polysaccharide capsule. Moreover, they may also secret different polysaccharides (known as exopolysaccharides) into the external environment. Representative exopolysaccharide structures of cepacian (produced by *B. cepacia*), alginate, and Psl (produced by *P. aeruginosa*) are shown in the inset. Mycobacteria **(right part)** contain a large cell wall complex formed by peptidoglycan, arabinogalactan, and mycolic acids of the so-called mycomembrane, and display other distinctive glycan structures, such as lipomannan, lipoarabinomannan, phosphatidyl-myo-inositol-mannosides, and trehalose mycolates. Sugar residues are depicted using the Symbol Nomenclature for Glycans (SNFG) (Varki et al., 2015; Neelamegham et al., 2019).

**FIGURE 2:**
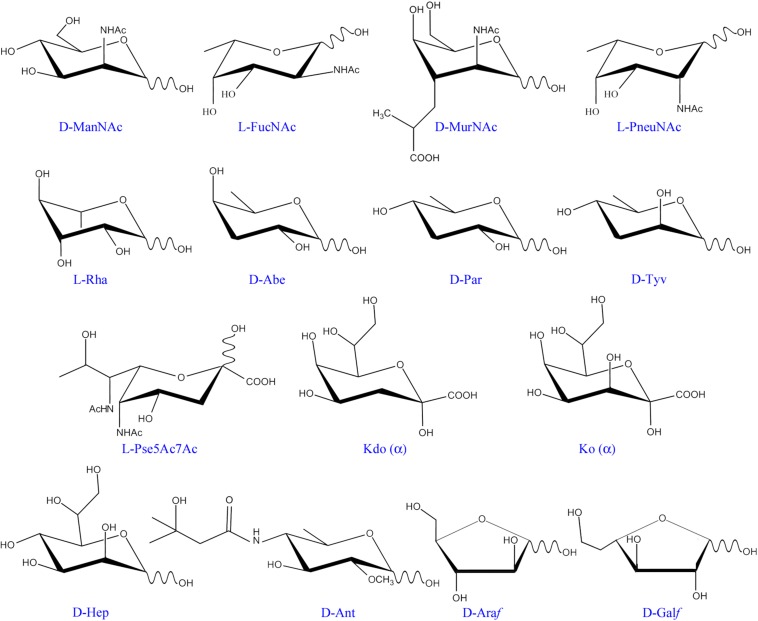
Monosaccharide residues found in bacteria, but not in mammals. Only those monosaccharides mentioned in the text have been included. ManNAc, *N*-acetyl-mannosamine; FucNAc, *N*-acetyl-fucosamine; MurNAc, *N*-acetyl-muramic acid; PneuNAc; *N*-acetyl-pneumosamine; Rha, rhamnose; Abe, abequose; Par, paratose; Tyv, tyvelose; Pse5Ac7Ac, 5,7-di-*N*-acetyl pseudaminic acid; Kdo, 3-deoxy-D-manno-oct-2-ulosonic acid; Ko, D-glycero-D-talo-oct-2-ulosonic acid; Hep, L-glycero-D-mannoheptose; Ant, anthrose; Ara*f*, arabinofuranose; Gal*f*, galactofuranose.

The intricate network of glycans covering bacterial surfaces differs between Gram-negative and Gram-positive bacteria (Figure 1) (Salton and Kim, 1996). Gram-negative bacteria are enveloped by two cell membranes separated by a thin peptidoglycan layer, and display lipopolysaccharides (LPSs) embedded in the outer membrane. LPSs are anchored to the membrane through a highly conserved lipid A moiety that is linked to a polysaccharide composed of an inner and outer core and an outermost chain built with repeating saccharide units, which is alluded to as O-chain or O-antigen (Figure 1, left part). Some Gram-negative bacteria, however, do not contain O-antigen chains in their LPS, which is therefore referred to as lipooligosaccharide (LOS). In contrast, Gram-positive bacteria only have one cell membrane that is covered by a thick peptidoglycan layer, and they usually display teichoic acids (TAs) anchored to the membrane (known as lipoteichoic acids or LTAs) or covalently bound to the peptidoglycan (known as wall teichoic acids or WTAs) (Figure 1, middle part). Common to several Gram-negative and -positive bacteria is the potential presence of cell surface glycoproteins and capsular polysaccharides. Mycobacteria can be considered apart from these two main groups as they display a unique envelope distinguished by a large cell wall complex formed by peptidoglycan covalently attached to arabinogalactan, which in turn is linked to long fatty acids (mycolic acids) that constitute the inner leaflet of the so-called mycomembrane (Figure 1, right part) (Jankute et al., 2015). Arabinomannan, lipoarabinomannan, phosphatidyl-myo-inositol-mannosides, phenolic glycolipids, and trehalose-containing lipids are other distinctive glycan structures of the mycobacterial envelope (Figure 1, right part). Overall, the repertoire of bacterial glycans shows a huge diversity in monosaccharide residues and linkage configurations, many of which are not found in the eukaryotic glycome (Figure 2) (Herget et al., 2008; Adibekian et al., 2011). The precise structure of these glycans may substantially differ among bacteria with the same cell surface architecture, and even among different strains of a given bacterial species. Moreover, some bacteria display very rare sugars, e.g., 3,6-dideoxyhexoses, which are found in a limited number of Enterobacteriaceae, or the 4,6-dideoxy sugar anthrose, distinctive of *Bacillus anthracis* (Figure 2). Thus, the specific glycans that decorate the bacterial surface can serve to typify strains.

Many bacterial glycans are immunogenic and have been used to develop vaccines against the respective bacteria. In addition, they may be recognized as “non-self” by host pattern-recognition receptors, including a variety of lectins of the innate immune system, for triggering defense mechanisms (Sukhithasri et al., 2013; Wesener et al., 2017; Casals et al., 2018). Not surprisingly, some bacteria camouflage from the host by displaying glycans that mimic the carbohydrate moieties of host cells. Moreover, recognition of such self-like glycans by host lectins may be exploited by the bacterium for down-regulating the innate immunity, or as stratagem for promoting attachment through lectin bridging of bacterial and host glycans. On the other hand, several bacteria bind directly to host glycans using surface-exposed adhesins (Moonens and Remaut, 2017), and in some cases these adhesins are also involved in the formation of bacterial microcolonies and biofilms through binding to glycans of neighbor cells or to secreted exopolysaccharides. Similarly, bacteriophages frequently target bacterial glycans for invading their hosts or to release the phage progeny. In addition, many bacterial hydrolases, e.g., those hydrolyzing the cell wall, are modular and contain carbohydrate-binding modules (CBMs) that bind to specific regions of the substrate to situate the catalytic domain at a position appropriate for cleavage. Furthermore, there is a specific class of plant receptors able to recognize bacterial oligosaccharides that operate as signaling molecules in plant−bacteria symbiosis. Thus, a broad variety of proteins from different life kingdoms recognize bacterial glycans and play important roles in the cross-talk of bacteria with their particular environment. Therefore, delineation of bacterial surface glycosignatures and assessment of their recognition by relevant glycan-binding proteins is crucial to understand, and when possible govern, the bacteria’s behavior. To this aim, different microarray approaches have been developed.

The microarray technology emerged to meet the scientist’s desire of a high-throughput analytical tool that enabled simultaneous analyses of a large number of biomolecular interactions using very small amounts of sample. The underlying concept was that a high local concentration of a given sample clustered in a miniature spot could enhance detection sensitivity. Prompted by the great success of DNA microarrays in gene expression profiling and related applications (Morley et al., 2004), protein (Zhu et al., 2001; Angenendt, 2005; Tao et al., 2008) and carbohydrate microarrays were also developed (Fukui et al., 2002; Blixt et al., 2004; Campanero-Rhodes et al., 2006), allowing high-throughput studies of protein expression and functionalities, including carbohydrate-mediated recognition events (Blumenschein et al., 2007; Campanero-Rhodes et al., 2007). Initially, most glycan libraries included in the arrays were mainly composed of mammalian-like structures, casting doubt on their value for exploring the binding preferences of proteins that recognize bacterial glycans. To overcome this limitation, growing efforts are being made to generate microarrays incorporating bacterial carbohydrate structures, ranging from small synthetic fragments to large natural polysaccharides.

**FIGURE 3:**
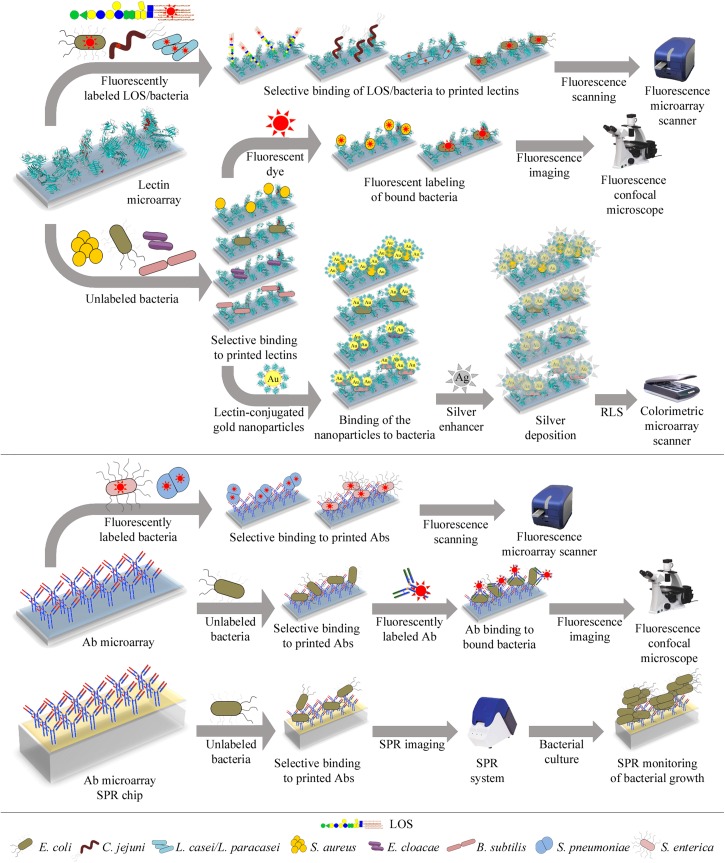
Illustration of different lectin and antibody microarray approaches used for glycophenotyping, differentiation, and detection of bacteria. **Top panel**: Microarrays containing a collection of lectins with diverse carbohydrate-binding specificities can be incubated with fluorescently-labeled bacteria or LOS and bound targets directly quantified using a fluorescence microarray scanner **(upper row)**. Alternatively, the microarrays can be incubated with unlabeled bacteria and bound bacteria next labeled with a fluorescent dye, enabling detection by confocal microscopy **(middle row)**. Bound unlabeled bacteria can also be detected by incubation with gold nanoparticles conjugated to a lectin known to recognize the bacteria under study. The resonance light scattering (RLS) of the nanoparticles is then enhanced by deposition of silver and next measured using a colorimetric microarray scanner **(lower row)**. **Bottom panel**: Microarrays containing antibodies (Abs) raised against selected bacteria can be incubated with fluorescently-labeled bacteria and bound bacteria detected by fluorescence scanning **(upper row)**. The microarrays can also be incubated with unlabeled bacteria, followed by incubation with fluorescently-labeled anti-bacteria Abs, and bound Abs are next detected by confocal microscopy **(middle row)**. Finally, bacteria selectively bound by Abs arrayed on SPR (surface plasmon resonance) chips can be detected by monitoring their growth during on-chip culture, using SPR imaging **(lower row)**. Specific bacteria that have been tested using these different approaches are detailed in each case.

**FIGURE 4:**
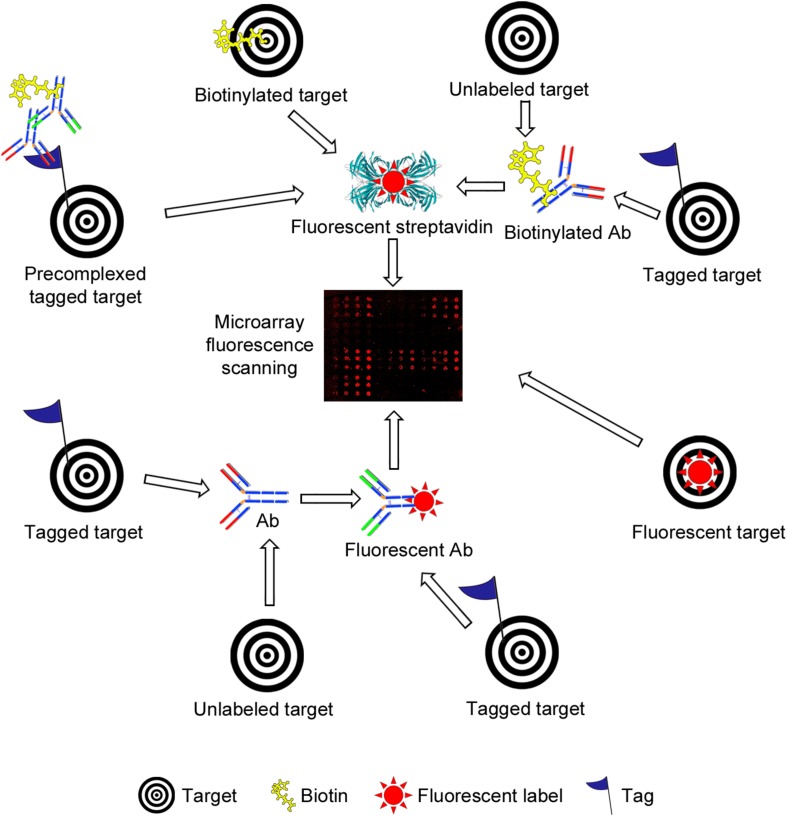
Schematic representation of different strategies used for fluorescence-based detection of lectin and antibody (Ab) binding to bacterial carbohydrate and whole cell microarrays. The simplest setup involves incubation with a fluorescently labeled target **(lower right side)**. A common strategy is the use of biotinylated targets, which are next detected by incubation with fluorescently labeled streptavidin **(upper part)**. The targets may carry other tags (e.g., His- or Fc-tags), and detection then has involved the use of biotinylated or unlabeled Abs, followed by incubation with streptavidin or with a biotinylated secondary Ab, as appropriate. Pre-complexing tagged targets with primary and secondary Abs has been exploited to reduce the number of incubation steps and/or to increase the sensitivity of detection. Alternatively, tagged targets have been detected by incubation with a fluorescent or unlabeled Ab, the latter followed by incubation with a fluorescent secondary Ab **(lower part)**. Finally, the binding of unlabeled targets has been monitored using biotinylated or unlabeled primary Abs followed by fluorescently labeled streptavidin or secondary Abs. In all cases, the final step involves the scanning of fluorescence signals.

Microarrays are frequently assembled on microscope glass slides coated or derivatized with a variety of reagents, depending on the nature of the samples to be immobilized (the probes) and the surface chemistry of choice (please see Tables 1, 2 for selected examples covered by this review). The binding of samples of interest (the targets) to the arrays is then assessed, typically using fluorescent detection systems that further enhance the sensitivity of the technique (Figures 3, 4), although other methods have also been used for detection (Figure 3 and Table 1).

**TABLE 1:** Lectin and antibody microarrays used for glycophenotyping, differentiation, and detection of bacteria.

**Printed probes**	**Slide surface chemistry^a^**	**Tested targets**	**Detection strategy**	**Detection technique**	**References**
16 Lectins	Epoxy activated	*E. coli*, *E. cloacae*, *S. aureus*, *B. subtilis*	Lectin-conjugated gold nanoparticles followed by silver deposition	Resonance light scattering	Gao et al., 2010
15 Lectins	NHS activated polyacrylamide hydrogel coating	*S. aureus*	SYTO 60 labeling of bound bacteria	Fluorescence confocal microscopy	Liu et al., 2016
21/41 Lectins	NHS activated multi-component hydrogel coating	*E. coli* (4 strains)/*C. jejuni* (2 strains)	Bacteria labeled with SYTO 85/SYTOX Orange	Fluorescence scanning	[Bibr B38][Bibr B46]
44 Lectins	Epoxysilane activated	*L. casei*, *L*. *paracasei* (16 strains)	Bacteria labeled with SYTOX Orange	Evanescent-field fluorescence scanning	Yasuda et al., 2011
8/15 Lectins + 2 Abs	Epoxy activated	*C. jejuni* LOS (3/8 strains)	LOS labeled with BODIPY	Fluorescence scanning	Semchenko et al., 2012a
ConA	ZnO nanorod arrays on fluorine-doped tin oxide glasses	*E. coli*	DAPI labeling of bound bacteria	Fluorescence microscopy	Zheng L. B. et al., 2017
3 Lectins + 3 sugars	Carbon nanotubes on gold electrodes	*E. coli* K12, *E. faecalis*, *S. mutans, S.* Typhi		Measurement of electronic resistance	Saucedo et al., 2018
Anti-*E. coli* O157:H7 Ab	Gold slides coated with biotin-labeled BSA + streptavidin (for printing of biotin-labeled Ab)	*E. coli* O157:H7	Fluorescein-labeled anti-*E. coli* O157:H7 Ab	Fluorescence microscopy	Gehring et al., 2006
6 Abs + 6 O-chain polysaccharides	Epoxy activated	*E. coli* (6 non-O157 STEC strains)	Alexa Fluor 555-labeled Abs	Fluorescence scanning	Hegde et al., 2013
7 Abs (pyrrole conjugates)	Gold-covered biochips (electrochemical arraying)	*E. coli* (15 STEC + 2 non-STEC strains)	Real-time monitoring of bacterial growth	SPR imaging	Mondani et al., 2016
35/66 Abs	Epoxy activated	*Salmonella enterica/Streptococcus pneumoniae*	Bacteria labeled with Eosin Y/SYTO25	Fluorescence scanning	[Bibr B14][Bibr B55]

*^*a*^Glass slides unless otherwise indicated; NHS, *N*-hydroxysuccinimide; SPR, surface plasmon resonance; Ab, antibody.*

This review gives different examples on the application of the microarray technology to explore bacterial surface glycans and their interactions with diverse glycan-binding proteins. Lectin and antibody microarrays have served to examine bacterial glycosignatures, facilitating bacteria identification and differentiation among strains, and to spot variations in glycan structures derived from changes in environmental conditions. In addition, they have been exploited for detection of bacteria in a diversity of samples, extending from sera to soils. Microarrays incorporating bacterial carbohydrate structures have proved to be useful for serodiagnosis of bacterial infections, identification of antigenic determinants for vaccine development, and mapping of epitopes recognized by bacteria-specific anti-carbohydrate antibodies. Moreover, they have served to identify bacterial ligands for lectins of the innate immune system and for bacterial and phagic proteins. Finally, microarrays of cell wall fragments and entire bacterial cells have been used to profile accessible carbohydrate structures on the bacterial surface, and to examine their interactions when they are displayed on the cell surface, thus preserving their natural arrangement, distribution, and density.

## Lectin Microarrays for Glycophenotyping of Bacteria

A diversity of lectin microarrays has been developed and applied to the analysis of the glycosylation profiles of different bacteria, also enabling differentiation among strains of a given bacterium, and to monitor variations in their glycosignatures associated with changes in culture conditions. These microarrays exploit the ability of lectins to selectively recognize specific carbohydrate structures on the bacterial surface. An example is the comparison of the binding patterns of *Escherichia coli* (laboratory strain DH5α), *Enterobacter cloacae*, *Staphyloccocus aureus* (Rosenbach), and *Bacillus subtilis* to an array of 16 lectins with various carbohydrate-binding specificities (Gao et al., 2010). A peculiarity of this study was the detection of bound bacteria using gold nanoparticles functionalized with *Griffonia simplicifolia* lectin II (GSL-II), which is specific for *N*-acetylglucosamine (GlcNAc, please see Table 3 for detailed information on the binding specificities of model lectins mentioned in this review) and was shown to recognize the four bacteria, followed by silver deposition to enhance the resonance light scattering of the particles, finally used for quantitation (Figure 3 and Table 1). Clearly different binding patterns were observed, with distinctive features for each bacterium. For example, whereas strong binding of *E. coli*, *E. cloacae*, and *B. subtilis* by the galactose (Gal)-specific agglutinins from *Ricinus communis* (RCA) and *Maackia amurensis* (MAA-I), and by the sialic acid-specific *M. amurensis* lectin II (MAA-II) was observed, this was not the case for *S. aureus*, whose binding pattern was additionally characterized by the intensity of the signal for the fucose-specific *Aleuria aurantia* lectin (AAL). On the other hand, *B. subtilis* was distinguished by the binding signals for the lectins from *Erythrina cristagalli* (ECL) and, specially, *Datura stramonium* (DSL), while *E. coli* gave the strongest signal among the four bacteria tested for soybean agglutinin (SBA). Interestingly, signal intensities and binding patterns of *E. coli* and *S. aureus* appeared to change when these bacteria were grown in different culture media, suggesting variations in their surface glycans. An intriguing result of this study was the low signal observed for the binding of *S. aureus* by wheat germ agglutinin (WGA), particularly when compared with GSL-II as WGA also recognizes GlcNAc (see Table 3). Indeed, a later study of these authors using different procedures for lectin immobilization and detection of bacterial binding (specified in Table 1) showed comparable binding signals of the same *S. aureus* strain for WGA and GSL-II (Liu et al., 2016). This discrepancy could tentatively be explained by a different binding activity of printed WGA, possibly derived from the immobilization strategies employed, and draws attention to the importance of using appropriate internal controls of lectin activity.

**TABLE 3:** Binding specificities of model lectins mentioned in this review.

**Lectin**	**Abbreviation**	**Source**	**Monosaccharide**	**Sugar-binding preferences**
Concanavalin A	ConA	*Canavalia ensiformis* seeds	Man/Glc	α-Methyl-mannopyranoside > α-Man > α-Glc > α-GlcNAc
Wheat germ agglutinin	WGA	*Triticum vulgaris*	GlcNAc	[GlcNAc]_3_, [GlcNAc]_2_, GlcNAc. It may bind Neu5Ac, but not Neu5Gc
*Griffonia simplicifolia* lectin II	GSL-II	*Griffonia simplicifolia* seeds	GlcNAc	Terminal α- or β-GlcNAc residues
*Datura stramonium* lectin	DSL	*Datura stramonium* seeds	GlcNAc	β(1-4)-linked GlcNAc oligomers: Chitotriose > chitobiose > GlcNAc. Also LacNAc and LacNAc oligomers
Peanut agglutinin	PNA	*Arachis hypogaea* peanuts	Gal	Galβ(1-3)GalNAc (T-antigen), Lac
*Ricinus communis* agglutinin	RCA-I, RCA_120_	*Ricinus communis* seeds	Gal	Terminal β-Gal. Galβ(1-4)Glc >> Galβ(1-3)Glc. GalNAc is a very poor inhibitor
*Viscum album* agglutinin	VAA	*Viscum album* leaves	Gal	Terminal β-Gal
*Artocarpus integrifolia* lectin	Jacalin	*Artocarpus integrifolia* seeds	Gal	Galβ(1-3)GalNAc (T-antigen), non-, mono- or di-sialylated
*Erythrina cristagalli* lectin	ECL	*Erythrina cristagalli* seeds	Gal	Lac, LacNAc
Soybean agglutinin	SBA	*Glycine max* seeds	GalNAc/Gal	Terminal α- or β-linked GalNAc and to a lesser extent Gal residues
*Helix pomatia* agglutinin	HPA	*Helix pomatia* albumin gland	GalNAc	α-GalNAc over β-GalNAc. Weakly α-Gal
*Aleuria aurantia* lectin	AAL	*Aleuria aurantia* mushrooms	Fuc	Fuc α(1-6)-linked to GlcNAc or α(1-3)-linked to LacNAc related structures
*Maackia amurensis* lectin I	MAL-I	*Maackia amurensis* seeds	Gal	Galβ(1-4)GlcNAc
*Maackia amurensis* lectin II	MAL-II	*Maackia amurensis* seeds	Neu5Ac	Neu5Acα(2-3)Galβ(1-3)GalNAc

*Man, mannose; Glc, glucose; GlcNAc, *N*-acetylglucosamine; Gal, galactose; GalNAc, *N*-acetylgalactosamine; Fuc, fucose; Neu5Ac, *N*-acetylneuraminic acid; Neu5Gc, *N*-glycolylneuraminic acid; LacNAc, *N*-acetyllactosamine; Lac, lactose.*

Besides providing information on the glycosylation profiles of different bacteria, lectin microarrays can aid to differentiate strains of a given bacterium. This was first demonstrated by Hsu et al. (2006), who compared the lectin binding fingerprints of two closely related K12 *E. coli* strains (defective in O-chain synthesis) and of *E. coli* RS218, a neonatal meningitis pathogen. Using a panel of 21 lectins, whose binding activity was verified with fluorescently-labeled glycoprotein standards, clear differences in binding patterns and intensities were observed. In particular, the two K12-derived strains showed strong binding by GSL-II, WGA, and the α-*N*-acetylgalactosamine (α-GalNAc)-specific lectin from the snail *Helix pomatia* (HPA), but only one of them gave meaningful binding signals with four other lectins, suggesting the presence of different repertoires of surface glycan structures. This was even more evident for the pathogenic *E. coli* strain, which gave positive signals with 10 lectins of the panel. Since the invasiveness of *E. coli* RS218 was known to be growth dependent, the possibility that the lectin binding patterns could also change with growth was examined. A general decrease in the intensities of all the positive signals was observed when progressing from the lag phase to the stationary phase (Hsu et al., 2006), suggesting a possible correlation of glycosylation with invasion. Overall, the approach proved to be useful for distinguishing *E. coli* strains and monitoring dynamic alterations in the cell surface glycans.

A similar approach was exploited to compare the lectin binding patterns of 16 *Lactobacillus casei/paracasei* strains indistinguishable from each other by 16S rRNA sequences (Yasuda et al., 2011). Using a panel of 44 lectins, a unique binding fingerprint was observed for 13 of these strains. Interestingly, half of the strains were bound by only one or two lectins, whereas the rest were recognized by multiple lectins with different carbohydrate-binding specificities, again pointing to a diversity of glycan structures. Thus, the assays enabled differentiation of strains, at the same time providing information on the carbohydrate determinants on the bacterial surface that are accessible for recognition.

*Campylobacter jejuni* is responsible for gastroenteritis in humans, while it is a commensal in chicken. A main difference between human and avian hosts is their core body temperature (37 and 42°C, respectively), what could be important for specific adaptation and ensuing pathogenesis or commensalism outcomes. In order to explore the effect of temperature on the surface glycans, two strains isolated from human hosts, the highly virulent *C. jejuni* 81-176 and the comparatively less invasive *C. jejuni* 81116, were cultured at 37 and 42°C and their lectin binding patterns were examined using a microarray comprising 41 lectins, whose binding activities were verified with fluorescently-labeled control glycoproteins (Kilcoyne et al., 2014). Distinctive hapten-inhibitable binding patterns for strains grown at 37°C were observed, being in general compatible with known structures of their surface glycans. For strain 81116 cultured at 42°C, an important decrease in the most intense binding signals was observed. These signals corresponded to lectins specific for Gal, lactose (Galβ(1-4)Glc), or GlcNAc, which are present in the LPS-like structure described in this strain, thus pointing to a decreased expression or alteration of this structure. In contrast, the changes in the lectin fingerprint of the virulent strain 81-176 grown at 42°C were more subtle, with only a subset of lectins showing small variations in binding intensity. This implied a relatively constant repertoire of glycan structures accessible for recognition. Based on the binding specificity of the lectins involved, these structures most probably include the capsular polysaccharide (CPS) and the LOS, which are known to play different roles in adhesion and invasion of epithelial cells as well as evasion of the immune system.

*Campylobacter jejuni* produces a variety of LOS structures that mimic mammalian gangliosides, what is thought to induce anti-ganglioside antibodies in the host and the subsequent development of neuropathies. A mixed lectin and antibody array was used to screen the LOS of *C. jejuni* strains for molecular mimicry (Semchenko et al., 2012a, b). First, a panel of 8 lectins, including cholera toxin subunit B (CTB), which binds ganglioside GM_1_, together with two antibodies against gangliosides GM_1_ and GM_2_ as positive controls, were used to examine LOS preparations from strains 11168-O (known to mimic GM_1_), 81-176 (GM_2_ like), and 224 (unknown LOS type) (Semchenko et al., 2012b). Surprisingly, none of the LOS was bound by CTB, what could be due to a loss of lectin activity upon incorporation into the array, again stressing the importance of verifying the activity of printed lectins. The LOS from strain 11168-O gave strong binding signals for the Gal-specific agglutinins from *Arachis hypogaea* (PNA), *Viscum album* (VAA), and *Artocarpus integrifolia* (jacalin), in agreement with the presence of terminal Gal as found in GM_1_, whereas a significantly lower binding by these lectins was observed for the LOS from strain 81-176, compatible with the absence of terminal Gal in its GM_2_-like structure. In comparison, although the LOS from strain 224 was bound by the anti-GM_1_ antibody, the binding signals for the Gal-specific lectins were equal to those observed for 81-176 rather than 11168-O. Using an extended array comprising 15 lectins, the binding patterns of the LOS from these three and five other uncharacterized *C. jejuni* strains were next examined (Semchenko et al., 2012a). Intriguingly, in this study no significant binding by VAA was observed for LOS 11168-O, indicating that, besides the procedure used for lectin printing, other factors, as, e.g., the activity of the specific lectin preparation employed, may affect the results. Based on the comparison of the antibody and lectin patterns of the uncharacterized LOSs with those of *C. jejuni* 11168-O- and 81-176-derived LOSs (with known GM_1_- and GM_2_-like structures, respectively), their terminal end structures were proposed. A parallel typing of LOS biosynthesis cluster, using a standard PCR method, revealed that the cluster type alone does not always allow prediction of the real LOS structure, highlighting the usefulness of the lectin microarray approach as complementary tool for evaluating the potential of clinical *C. jejuni* isolates to induce adverse autoimmune reactions.

The capture of bacteria by lectin arrays can also be exploited for detection of pathogenic bacteria in the clinical field as well as in the environmental or agri-food sectors. A ZnO nanorod array functionalized with concanavalin A (ConA), a mannose (Man)/glucose (Glc)-specific lectin from the legume *Canavalia ensiformis*, was employed for capturing *E. coli* (Table 1) and proved to work efficiently with reasonable detection limits and linear range (1.0 × 10^3^ to 1.0 × 10^7^ cfu mL^–1^) even in complex samples, so it could be applied to the analysis of real samples (Zheng L. B. et al., 2017). More recently, a lectin and saccharide nano-chemiresistor array was used to detect *E. coli* K12, *Enterococcus faecalis, Streptococcus mutans*, and *Salmonella enterica* sv. Typhi (Saucedo et al., 2018). The array consisted in carbon nanotubes assembled on the surface of gold electrodes and functionalized with three lectins (ConA, PNA, and WGA) and three aminophenyl saccharides (Gal, Glc, and Man). After incubation with bacteria, changes in the electronic properties were monitored by measuring device resistance. *E. coli* and *S.* Typhi, both Gram-negative bacteria, gave noticeably different patterns, whereas for Gram-positive *E. faecalis* and *S. mutans* the patterns were more similar, although still clearly distinguishable. Detection was achieved at clinically relevant concentrations, indicating that an array with carefully chosen probes could be used as diagnostic tool.

## Antibody Microarrays for Detection and Serotyping of Bacteria

Bacterial surfaces display a variety of antigens that can be used for identification and typing of antigenically distinct strains. Different microarrays incorporating O-antigen- or capsular-specific antibodies have been used to this aim. One example is the detection of Shiga-toxin producing *E. coli* (STEC), which is frequently identified as the pathogen responsible for food-illnesses and causes severe enteric infections such as diarrhea, hemorrhagic colitis, or even hemolytic uremic syndrome, a life-threatening complication. *E. coli* O157:H7 was the first enterohemorrhagic *E. coli* serotype detected in an outbreak in United States provoked by the consumption of contaminated burgers. The potential of antibody microarrays for detecting the bacterium was put forward by Gehring et al. (2006), who used a polyclonal anti-*E. coli* O157:H7 antibody printed onto microarray slides (Table 1) for capturing *E. coli* O157:H7 cells, in turn detected with fluorescently-labeled anti-*E. coli* O157:H7 antibody (sandwich fluorescent immunoassay, see Figure 3). A linear fluorescent response was observed from ∼3.0 × 10^6^ to ∼9.0 × 10^7^ cells/mL. A similar sandwich immunoassay was later used for identification of six other STEC serogroups, i.e., O26, O45, O103, O111, O121, and O145 (the top six non-O157 serogroups), which have been associated with 70–80% of non-O157 STEC-produced illnesses. Microarrays incorporating antibodies specific for one of these six O-antigens, along with the respective O-antigen polysaccharides as positive control, were tested for the binding of reference strains belonging to these serogroups and found to yield specific and reproducible signals at bacterial concentrations of 10^6^ CFU/mL and above (Hegde et al., 2013). STEC can represent a serious threat to human health at very low contaminating levels (less than 100 CFU per sample), far below the limits of detection of this microarray approach and of other techniques commonly used. Consequently, a pre-enrichment step is always required. Several foods have been identified as potential sources of STEC, including tap water, but the main source and reservoir is beef. Indeed, a recent outbreak in United States has been associated with the consumption of ground beef contaminated with *E. coli* O103^1^. Therefore, the efficiency of the antibody microarray for serotyping contaminant non-O157 STEC in food was evaluated by testing ground beef samples enriched for 12 h after inoculation with 1–10 CFU of target serogroups, alone or in combination with one or two other serogroups (Hegde et al., 2013). All target groups were identified with no cross reactions, supporting the usefulness of the approach for the simultaneous detection of different STEC serogroups.

An antibody microarray approach for fast detection of O157 and the top six non-O157 STEC serogroups without the need of a pre-enrichment step was reported by Mondani et al. (2016). The approach was based on the on-chip culture of bacteria captured by the arrayed antibodies (Figure 3), and real-time monitoring of bacterial growth by surface plasmon resonance imaging (SPRi), a method previously found to be efficient for detecting *E. coli* O157:H7 at very low initial concentrations (Bouguelia et al., 2013; Mondani et al., 2014). Fifteen different strains belonging to the seven STEC serogroups, plus two non-STEC strains, were analyzed on SPR biochips presenting electrochemically arrayed antibodies against the target serogroups (Table 1). All STEC serogroups were successfully identified, even at initial concentrations in the range encountered in naturally contaminated samples (few CFU ml^–1^), and no response was observed for the non-STEC strains. Moreover, *E. coli* O157:H7 was successfully detected in ground beef artificially contaminated with only few cells (5 CFU per 25 g). Thus, considering that detection of bacteria is carried out during enrichment, thereby reducing the processing time, the approach could be a faster alternative to other methods commonly used for detection of STEC in contaminated food.

Antibody microarrays have also proved to be useful for high-throughput serotyping of bacteria. As example, microarrays incorporating antisera against selected *Salmonella* O and H (flagellar proteins) antigens were efficient for serotyping *S. enterica* strains (Cai et al., 2005). Using 117 target strains, belonging to the top 20 commonly isolated and clinically relevant serotypes, and 73 non-target strains, this microarray approach successfully allowed one-step full or partial identification of 86 and 30 target strains, respectively, and exclusion of all non-target strains.

In the case of *Streptococcus pneumoniae*, the capsule is one of the major pathogenicity factors. Currently, 98 different serotypes, divided into 25 individual types and 21 serogroups, composed of two to eight serotypes with related capsular antigenic determinants that can be differentiated using factor (individual capsular antigen) antisera, have been identified. A microarray containing 66 different group-, type- and factor-specific antisera, with specificity for 83 of the 98 *S. pneumoniae* serotypes, was first tested with *S. pneumoniae* reference isolates of these 83 serotypes and found to correctly serotype 94% of the samples. Only 11 isolates within the same group were mistyped and for four samples a detectable signal was not obtained (Marimon et al., 2010). To test the utility of the microarray in clinical practice, 226 *S. pneumoniae* clinical isolates (106 invasive isolates and 120 randomly-selected non-invasive isolates) were next examined, in direct comparison to serotyping by latex agglutination followed by the Quellung reaction. Only for 7.1% of the isolates discrepant serotyping by the two methods was found. Moreover, for these isolates, PCR amplification of each capsular gene showed that only one isolate was misidentified by the microarray. Thus, the microarray approach proved to be an accurate serotyping technique and could be a valuable tool for pneumococcal epidemiological studies.

## Bacterial Glycan Arrays for Serodiagnosis of Bacterial Infections

Exposure to bacterial antigens often induce the production of antibodies. A seminal study of Wang et al. (2002) demonstrated the usefulness of microbial glycan microarrays for detecting the presence in human serum of antibodies against several bacteria. An array incorporating a collection of carbohydrate-containing macromolecules, including 21 bacterial polysaccharides, was incubated with 1-μl human serum samples from normal individuals, and IgG and IgM antibodies captured in the array were independently detected using the respective anti-human IgG/IgM secondary antibodies (see Figure 4 for a schematic representation of different strategies used for fluorescent detection of target binding to bacterial carbohydrate microarrays). IgM binding to pneumococcus type 27 and different *Klebsiella* polysaccharides was spotted, and the repertoire of bacterial polysaccharides recognized by IgG antibodies was broader, also including *E. coli* types K92 and K100, group B meningococcus, *Haemophilus influenzae* type A, and 5 different pneumococcus types. These results questioned the traditional belief that naturally occurring anti-polysaccharide antibodies were mainly of IgM type, and demonstrated that the proposed system was efficient for detecting specific antibodies in human serum. Moreover, a microarray containing a panel of nine LPS preparations isolated from different bacteria, including *Francisella tularensis*, was later found to be efficient for detecting anti-*F. tularensis* LPS antibodies in tularemia-positive canine serum samples (Thirumalapura et al., 2005), while more focused arrays containing capsular and O-antigen saccharides from different strains of *Burkholderia mallei* and/or *Burkholderia pseudomallei* successfully detected specific antibodies in the serum of human patients infected with these bacteria (Parthasarathy et al., 2006, 2008). Altogether, these studies revealed the potential of bacterial glycan microarrays for the serological diagnosis of bacterial infections.

Different types of bacterial glycans have been included in the arrays (Table 2), essentially depending on the specific bacterium under study. For example, several *Salmonella* serogroups are characterized by displaying O-antigens containing 3,6-dideoxy-D-ribo- (paratose, abbreviated Par, serogroup A), -D-xylo- (abequose, abbreviated Abe, serogroup B), or -D-arabino- (tyvelose, abbreviated Tyv, serogroup D) hexose residues (Figure 2), α(1-3)-linked to a common Manα(1-4)Rhaα(1-3)Gal main chain (Rha standing for rhamnose). A microarray including synthetic di-, tri-, and tetrasaccharide glycosides based on these regions was tested with group-specific anti-*Salmonella* rabbit sera, showing a rather selective IgG binding to the respective O-antigens (Blixt et al., 2008). Based on the high specificity observed for the disaccharides Tyvα(1-3)Manα, Parα(1-3)Manα, and Abeα(1-3)Manα, their ability to detect *Salmonella*-specific antibodies in the serum of patients infected with *S. enterica* sv. Enteritidis (serogroup D) or *S. enterica* sv. Typhimurium (serogroup B) was examined in comparison to healthy controls. The first group of patients showed significantly elevated levels of antibodies against Tyvα(1-3)Manα, whereas the second group showed high reactivity toward Abeα(1-3)Manα, in both groups Parα(1-3)Manα giving only background signals. Therefore, O-antigen specific microarrays could be a suitable tool for serodiagnosis of *Salmonella* infections.

**TABLE 2:** Bacterial carbohydrate microarrays used for detection of bacteria-specific anti-carbohydrate antibodies and for recognition studies targeting diverse glycan binding proteins.

**Printed probes**	**Immobilization strategy**	**Slide surface chemistry^a^**	**Tested targets**	**References**
Synthetic structures of *C. difficile* CPS	Probes equipped with amino-linker	NHS activated	Human/mice sera, human feces^b^, hybridoma supernatant	Oberli et al., 2011 Martin et al., 2013b Broecker et al., 2016a
Polysaccharides and synthetic structures of *S. pneumoniae* or carbapenem-resistant *K. pneumoniae* CPSs	Probes equipped with amino-linker	NHS activated	Human/mice/rabbit sera, mAbs	Geissner et al., 2016 Parameswarappa et al., 2016 Emmadi et al., 2017 Lisboa et al., 2017 Menova et al., 2018 Seeberger et al., 2017 Menova et al., 2018 Diago-Navarro et al., 2018
Synthetic structures of *M. tuberculosis* CPS arabinomannan	Probes coupled to BSA	Epoxy activated	Human/mice sera	Chen et al., 2016 Prados-Rosales et al., 2017
Library of bacterial CPSs	Direct adsorption of unmodified probes	Nitrocellulose	Human sera	Wang et al., 2002
*S. enterica* O-chains and synthetic substructures	Probes equipped with amino-linker	NHS activated	Rabbit *Salmonella* typing sera, human sera	Blixt et al., 2008
Library of LPS O-chains + core	Unmodified probes or equipped with amino-linker	NHS activated	Langerin, galectins 3, 4, 8, 9, Gp047	Feinberg et al., 2011 Stowell et al., 2014 [Bibr B47][Bibr B40]
Library of synthetic LOS inner core structures	Probes equipped with amino-linker	Adipic acid dihydrazide-modified NHS activated	Human/mice sera, SP-D	Reinhardt et al., 2015 Reinhardt et al., 2016
Library of LPSs	Direct adsorption of unmodified probes	Nitrocellulose	Canine sera	Thirumalapura et al., 2005
Synthetic *M. tuberculosis* ManLAM or lipomannan structures	Probes equipped with thiol-linker	Maleimide-functionalized gamma amino propyl silane	Anti-ManLAM mAb, DC-SIGN	Chan et al., 2015 Leelayuwapan et al., 2017
Synthetic *C. difficile* LTA substructures	Probes equipped with amino-linker	NHS activated	Human sera, human feces^b^	Martin et al., 2013a Broecker et al., 2016b
Synthetic glycerol-based TA oligomers	Probes equipped with 2-aminobenzoic acid	Epoxy activated	Anti-*S. epidermidis* mAb, rabbit sera	van der Es et al., 2018
Synthetic peptidoglycan fragments	Probes equipped with amino-linker	Amorphous carbon with carboxylic acid surface	Peptidoglycan recognition protein PGRP-S	Wang et al., 2016
Natural and synthetic Nod factors, chitin oligosaccharides, and peptidoglycan-related compounds	Probes equipped with N-(2-aminoethyl)-4- (aminooxymethyl)benzamide linker	NHS activated	P60 autolysin, synthetic LysM domain	Maolanon et al., 2014 Sorensen et al., 2014
Library of glucan polysaccharide fragments	Neoglycolipids prepared by conjugation of probes to the aminolipids ADPH (reductive amination) or AOPE (oxime ligation)	Nitrocellulose	Anti-glucan mAbs, Dectin-1 DC-SIGN, DC-SIGNR, bacterial CBMs	Palma et al., 2006 Palma et al., 2015 Zhang et al., 2016 Li and Feizi, 2018
Cyclic β(1-2)-glucans	Probes embedded in a 3D matrix of a photoactive terpolymer	Nitrocellulose	DC-SIGN	Zhang et al., 2016
Synthetic fragments and derivatives of the tetrasaccharide of glycoprotein BclA from *B. anthracis* spores	Unmodified probes or equipped with thiol-linker	Photoactive phthalimide chromophores or maleimide-functionalized	Anti-*B. anthracis* spore Abs, anti-di/tetrasaccharide mAbs, cattle sera	Wang et al., 2007 Oberli et al., 2010 Tamborrini et al., 2011
*Burkholderia pseudomallei* CPS and LPS O-chain + core	Probes converted to glycosylamines by reductive amination	Epoxy activated	Human sera	Parthasarathy et al., 2006 Parthasarathy et al., 2008
Natural and synthetic *M. tuberculosis* polysaccharides	Unmodified probes or coupled to BSA	Epoxy activated	Human sera	Tong et al., 2005
*M. tuberculosis* lipid-linked glycans and polysaccharides	Unmodified probes	Nitrocellulose	Human ZG16p lectin	Hanashima et al., 2015
Library of synthetic *M. tuberculosis* representative structures	Probes equipped with amino-linker and coupled to BSA	Epoxy activated	DC-SIGN, DC-SIGNR, Dectin-2, langerin, MGR, mannose receptor, mincle	Zheng R. B. et al., 2017
Library of diverse synthetic bacterial structures	Probes equipped with amino- or thiol-linker	NHS/epoxy activated or maleimide-functionalized	MAbs, human sera, DC-SIGN, *B. cenocepacia* lectins A and C-Ct^c^	Geissner et al., 2019
Library of bacterial PSs, CPSs, and LPSs	Unmodified probes or equipped with amino-linker	NHS activated	Human sera, mice/rabbit Abs, galectins 3, 4, 8, langerin, intelectin-1	Stowell et al., 2014 Wesener et al., 2015 Hanske et al., 2017

*^*a*^Glass slides unless otherwise indicated; ^*b*^Supernatant of human feces; ^*c*^C-terminal domain of *B. cenocepacia* lectin C; NHS, *N*-hydroxysuccinimide; ADPH, *N*-aminoacetyl-*N*-(9-anthracenylmethyl)-1,2-dihexadecyl-*sn*-glycero-3-phosphoethanolamine; AOPE, 1,2-dihexadecyl-*sn*-glycero-3-*p*hospho*e*thanolamine; MGR, macrophage galactose receptor; Ab, antibody.*

**FIGURE 5:**
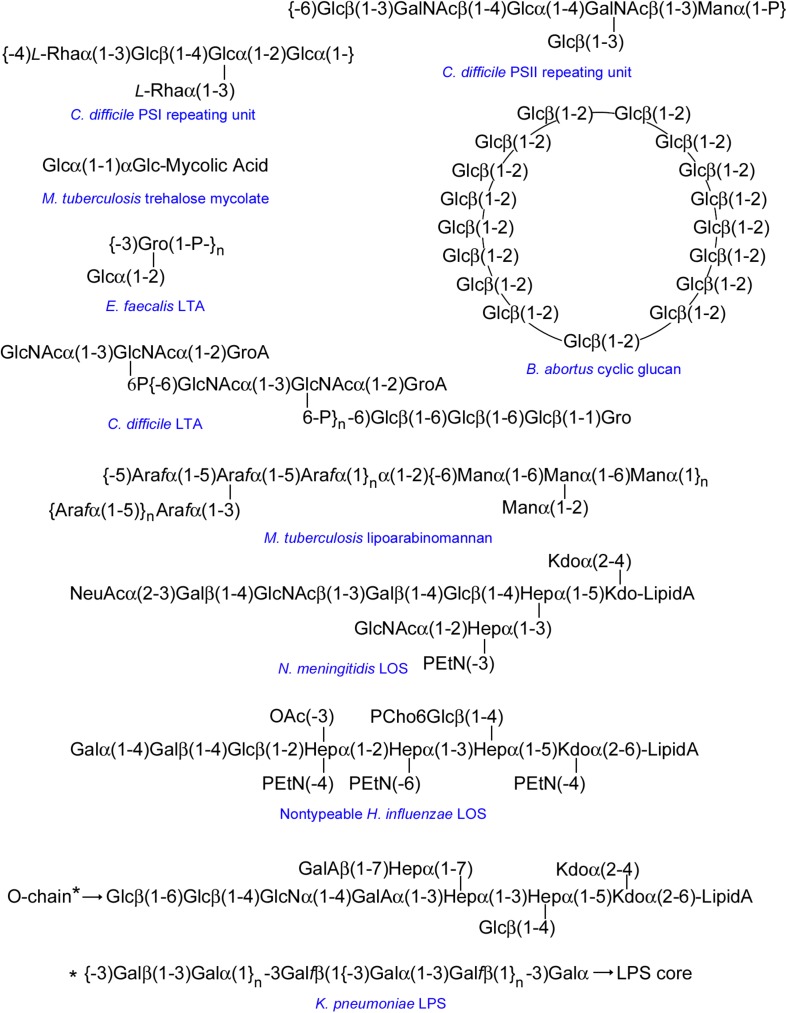
Selected examples of representative structures printed in bacterial carbohydrate microarrays. The repeating unit of *Enterococcus faecalis* LTA is shown as representative of a glycerol phosphate LTA backbone. Bacterial β(1-2)-linked cyclic glucans can occur in unsubstituted form or substituted at Glc C6 with anionic groups (e.g., succinyl in *B. abortus*). The structure shown for NTHi LOS corresponds to the major glycoform of strain 375. Because of space limitations, the structure of the O-chain of *K. pneumoniae* LPS is shown below the structure of the LPS core. Unless specifically indicated, sugar units are D-stereoisomers in pyranoside form. Sugars in furanoside form are labeled with the *f* suffix. GroA, glyceric acid; Gro, glycerol; GalA, galacturonic acid; P, phosphate; PEtN, phosphoethanolamine; OAc, *O*-acetyl; PCho, phosphorylcholine.

Mycobacteria display surface glycoconjugates very different from those of most other bacteria (Figure 1 right part). Tong et al. (2005) developed a multiplexed assay for serodiagnosis of tuberculosis based on a microarray containing 54 antigens of different classes, i.e., fractions of *Mycobacterium tuberculosis* cells and culture fluid, oligosaccharides conjugated to bovine serum albumin (BSA), purified LPSs and polysaccharides, and recombinant antigens. The goal was to identify antigens, or combinations thereof, allowing discrimination between culture-positive pulmonary tuberculosis patients, culture-negative patients with other pulmonary diseases, and healthy individuals. The authors found that a BSA conjugate containing the branched structure Araβ(1-2)Araα(1-3)[Araβ(1-2)Araα(1-5)]Araα(1-5)Ara of the cell wall glycolipid lipoarabinomannan (LAM, Figure 5), on its own, discriminated with good specificity and sensitivity between tuberculosis and non-tuberculosis sera, pointing out the applicability of LAM in serological tests.

## Bacterial Glycan Arrays for Identification of Novel Vaccine Candidates

Development of efficient vaccines to prevent bacterial infections can be facilitated by microarray-assisted identification of bacterial structures inducing an immune response and analysis of the specific epitopes recognized by vaccine-elicited protective antibodies. In tuberculosis patients, for example, antibody responses to LAM and to the related capsular polysaccharide arabinomannan (AM) correlate strongly, suggesting that AM is the immunogenic portion of LAM. A microarray containing a panel of 12 synthetic AM fragments, coupled to BSA, was used to assess the reactivity of IgG antibodies in the sera of 30 healthy *M. tuberculosis*-uninfected adults before and after primary or secondary vaccination with the licensed bacillus Calmette-Guerin (BCG) vaccine (Chen et al., 2016). In both vaccination groups, sera obtained 4 and 8 weeks after vaccination had significantly higher levels of AM-specific IgGs, although heterogeneous binding patterns to the microarray-printed AM fragments were observed. Interestingly, increased IgG titers correlated with enhanced BCG phagocytosis, particularly with IgG reactivity to three particular AM epitopes that contained at least two Man residues. Overall, the results suggested that AM-specific IgGs contribute to the defense against mycobacterial infection in humans. Moreover, immunization with AM-protein conjugates was also found to contribute to protection against infection (Prados-Rosales et al., 2017). In detail, immunization of mice with a 20 kDa AM fraction conjugated to *M. tuberculosis* Ag85b or to protective antigen from *B. anthracis* resulted in elevated levels of AM-specific antibodies able to stain *M. tuberculosis* cells, as observed by electron microscopy. To gain insight into the AM epitopes recognized by the antibodies, the binding of immune sera to a microarray including 30 BSA-conjugated synthetic AM fragments (representative of the mannan backbone, branched arabinan, and terminal Man residues) was examined. Binding to a diversity of fragments was observed, the most prevalent being linear or branched arabinan structures. Importantly, immunized mice next infected with the bacterium had lower bacterial loads in lungs and spleen and lived longer than control mice, with a marked reduction in mycobacterial dissemination. Thus, the humoral arabinan-targeted response elicited by the AM-protein conjugates can importantly contribute to the outcome of mycobacterial infection, suggesting that AM could be a good candidate for developing new vaccines against *M. tuberculosis*.

The glycan chain of the *B. anthracis* exosporium glycoprotein BclA, which decorates the surface of *B. anthracis* spores, also contains a unique tetrasaccharide structure consisting of 2-*O-*methyl-4-(3-hydroxy-3-methylbutamido)-4,6-dideoxy-Glc (termed anthrose and abbreviated Ant, Figure 2) β(1-3)-linked to Rhaα(1-3)Rhaα(1-2)Rha. A microarray including synthetic fragments and derivatives of this tetrasaccharide was examined for the binding of pooled rabbit polyclonal anti-anthrax spore IgG antibodies, revealing the presence of antibodies binding to anthrose-containing tri- and tetra-saccharides (Wang et al., 2007). Thus, the glycan chain of BclA appeared to be immunogenic and could be employed to develop novel vaccines targeting anthrax spores. In fact, mice immunization with the tetrasaccharide or with Antβ(1-3)Rha was later proved to elicit an antibody response, enabling the generation of monoclonal IgGs (Oberli et al., 2010). The binding specificity of several anti-tetrasaccharide and anti-disaccharide monoclonal antibodies (mAbs) was examined by microarray screening using a series of synthetic mono- to tetrasaccharides equipped with different anthrose side chain appendages. The anti-disaccharide mAbs recognized all the structures with intact anthrose, including anthrose monosaccharides, whereas the anti-tetrasaccharide mAbs required at least two Rha units as well as the terminal anthrose for tight binding. Although small modifications of the anthrose side chain only significantly affected anti-tetrasaccharide mAb binding, a drastic chain truncation abolished binding for all mAbs. Altogether, the results demonstrated that anthrose is the primary recognition unit. Interestingly, an anthrose-deficient *B. anthracis* lineage was identified in cattle from West Africa (Tamborrini et al., 2011), where anthrax is highly endemic and the majority of vaccines for cattle are based on live spores from an anthrose-positive strain. Thus, the spread of anthrose-deficient strains in this region could be an escape strategy of *B. anthracis*.

Microarrays containing synthetic structures based on TAs have also proved to be efficient for detecting anti-TA antibodies in serum (Table 2). A library of compounds comprising the most common glycerol phosphate backbone with 15 monomers in length, decorated by α-Glc, α-GlcN (glucosamine) or α-GlcNAc residues at various positions of the main chain (Figure 5), was interrogated for the binding of a mouse anti-*Staphylococcus epidermidis* mAb, serum obtained from rabbits immunized with *E. faecalis* LTA, and rabbit serum raised against a BSA-TA conjugate (van der Es et al., 2018). Clearly different IgG/IgM binding patterns were observed, unveiling selective recognition of specific TA epitopes and posing that TA-based vaccination strategies could be possible. Indeed, the potential of LTA glycans as vaccine candidates to protect from *Clostridium difficile* infections was previously proposed. This bacterium contains an unusual LTA phosphodiester-linked repeating unit with the sequence -6)GlcNAcα(1-3)[P6]-GlcNAcα(1-2)GroA (GroA being glyceric acid) (Figure 5). A microarray-printed synthetic dimer of this repeating unit was used to assess the binding of IgG antibodies in the serum of *C. difficile*-infected patients, unveiling recognition in six out of 12 tested samples and thereby suggesting that this epitope could be a relevant *C. difficile* antigen (Martin et al., 2013a). In a later study (Broecker et al., 2016b), a conjugate of the dimer and the carrier protein CRM_197_, a constituent of licensed vaccines, was used to immunize mice, and antibody responses in serum were followed using microarrays containing the dimer as well as monomers of the repeating unit with one or two phosphorylated GlcNAc residues. The results revealed that the conjugate elicited anti-LTA antibodies for which the minimal epitope for recognition was the repeating unit. Importantly, sera of immunized mice significantly opsonized all *C. difficile* strains and clinical isolates investigated. Moreover, colonization by *C. difficile* in immunized mice orally challenged with live bacteria was reduced compared with control mice. Thus, *C. difficile* LTA glycans emerged as potential vaccine candidates.

Two different *C. difficile* CPSs, named PS-I and PS-II, were also found to be antigenic and immunogenic. Both CPSs are present in a hypervirulent *C. difficile* strain responsible for outbreaks in North America and Europe. PS-I has a branched pentaglycosyl phosphate repeating unit [-4)Rhaα(1-3)Glcβ(1-4)[Rhaα(1-3)]Glcα(1-2)Glcα(1-P], while PS-II has a branched hexaglycosyl phosphate repeating unit [-6)Glcβ(1-3)GalNAcβ(1-4)Glcα(1-4)[Glcβ(1-]GalNAcβ(1-3)Manα(1-P] (Figure 5) (Ganeshapillai et al., 2008). First, the non-phosphorylated PS-II hexasaccharide was synthesized, conjugated to CRM_197_, and used to immunize mice (Oberli et al., 2011). Binding assays to the microarray-printed hexasaccharide showed the presence of specific IgG antibodies in the serum of immunized mice, indicating that the hexasaccharide was immunogenic. Moreover, specific IgA antibodies were detected in the feces of patients with *C. difficile* infection (Oberli et al., 2011; Martin et al., 2013b), suggesting that PS-II could be an antigenic determinant in humans. The non-phosphorylated PS-I pentasaccharide, together with mono-, di-, and tri-saccharide substructures thereof, were also synthesized and used for microarray screening of specific IgAs in feces and IgGs in serum of *C. difficile*-infected patients, in comparison to other patients and healthy controls. Variable antibody levels were detected in all groups, indicating that these structures represent biologically relevant epitopes. The main antigenic determinant of the pentasaccharide was explored by examining the binding to the arrays of sera of mice immunized with a PS-I pentasaccharide-CRM_197_ conjugate and of mAbs generated from such sera using the hybridoma technique (Broecker et al., 2016a), revealing that the disaccharide Rhaα(1-3)Glc, which is found twice in the pentasaccharide, is a minimal size epitope. Therefore, a simple disaccharide could be a valid target for developing novel vaccination approaches against *C. difficile*. Compared to the disaccharide, a construct displaying five disaccharide units showed noticeably tighter binding (about five orders of magnitude) to the mAbs and elicited in mice an IgG response more specific for larger glycans (Broecker et al., 2016a), thus limiting cross-reaction with structurally related glycans.

The antigenic CPS determinants of different *S. pneumoniae* serotypes were also investigated using a similar strategy, i.e., synthesis of the repeating unit and substructures thereof, construction of microarrays incorporating these synthetic structures, and screening of relevant sera for detection of recognized structures, often complemented with immunization of mice or rabbits with CRM_197_-conjugates of selected structures and subsequent microarray-assisted evaluation of serum antibodies and mAbs. Clearly distinct determinants were identified in each case. Thus, in serotype 2 the GlcAα(1-6)Glcα(1-2) branch (GlcA being glucuronic acid) was found to be an important substructure of the hexasaccharide repeating unit (Emmadi et al., 2017), while in serotype 7F the two side chains that decorate the linear tetrasaccharide backbone, i.e., Galβ(1- and GlcNAcα(1-2)Rhaα(1-, played a key role (Menova et al., 2018). Gal modification with a pyruvate ketal in the linear tetrasaccharide unit of serotype 4 was observed to be an important determinant, although pyruvate-independent epitopes were also unveiled (Geissner et al., 2016), whereas in the serotype 5 pentasaccharide unit the rare aminosugar *N*-acetyl-L-pneumosamine (PneuNAc, Figure 2) together with branched *N*-acetyl-L-fucosamine (FucNAc) were essential for antibody recognition and avidity (Lisboa et al., 2017). These findings could be of relevance for designing efficient synthetic glycoconjugate vaccines against *S. pneumoniae*.

In contrast to the above listed serotypes containing tetra- to hexasaccharide units, the repeating unit of *S. pneumoniae* serotype 3 CPS consists only of a disaccharide. Therefore, in this case, besides the respective disaccharide and monosaccharide units, one tetrasaccharide (comprising two repeating units) and two different trisaccharides with shifted sequences were synthesized and used in the microarray screening of two mAbs raised against serotype 3 CPS (Parameswarappa et al., 2016). The results showed that the tetrasaccharide was bound better than the smaller structures. Moreover, a tetrasaccharide-CRM_197_ conjugate was found to elicit opsonophagocytic antibodies in mice and confer protection against serotype 3 in a model of pneumococcal pneumonia (Parameswarappa et al., 2016), thus validating the usefulness of the approach.

The CPS of carbapenem-resistant *Klebsiella pneumoniae* has also been explored for their antigenic potential using a similar strategy. A CRM_197_-conjugate of the hexasaccharide repeating unit proved to be immunogenic in mice and rabbits, and elicited antibodies able to promote phagocytosis of the bacterium (Seeberger et al., 2017). CPSs have also been used to develop vaccines against different invasive serogroups of *Neisseria meningitidis*. However, in the case of meningococcal serogroup B, vaccines based on non-capsular antigens are needed because its capsule consists of autoantigenic α(2-8)-linked polysialic acid. As an alternative, the antigenic potential of the inner core structure of *N. meningitidis* LOS (Figure 5) was examined (Reinhardt et al., 2015). A library of species-specific mono- to tetrasaccharide structures was synthesized and used for microarray-assisted screening of human sera. Strong IgG binding to the tetrasaccharide GlcNAcα(1-2)Hepα(1-3)Hepα(1-5)Kdoα (Hep denoting L-glycero-D-mannoheptose, and Kdo denoting 3-deoxy-D-manno-oct-2-ulosonic acid), which is the conserved LPS inner core structure of all *N. meningitidis* immunotypes, and to the related trisaccharide lacking Kdo was observed, while binding to Hepα(1-3)Hepα(1-5)Kdoα was only weak, revealing the importance of the distal GlcNAc for recognition. Immunization of mice with a tetrasaccharide-CRM_197_ conjugate elicited an antibody response against the tetrasaccharide. Of note, mice serum antibodies bound to cells of a broad collection of *N. meningitidis* strains, and the binding to a LPS-free mutant was significantly lower, demonstrating the accessibility of the LPS inner core on the cell surface. Interestingly, epitope mapping using the microarray-printed library of synthetic structures revealed that, unlike human serum antibodies, Kdo was the immuno-dominant residue for the mice antibodies. A possible explanation posed by the authors is the presence in mouse germline antibodies of an inherited binding pocket specific for Kdo. In that case, mice might not be the best model to evaluate the synthetic Kdo-containing tetrasaccharide as potential vaccine candidate. Moreover, it is likely that in this structure the Kdo residue is much more exposed than in *N. meningitidis* cells, shed membrane vesicles, or fragments from opsonized bacteria that predictably elicited the antibodies detected in human serum.

## Bacterial Glycan Arrays for Testing Antibodies With Diagnostic or Therapeutic Potential

Besides aiding in the identification of vaccine candidates, bacterial glycan microarrays have helped to dissect the binding specificities of mAbs obtained for diagnostic or therapeutic purposes. An example is the antibody-based detection of tuberculosis biomarkers, which can form the basis of an inexpensive point-of-care diagnostic test. A suitable biomarker is the Man-capped form of LAM that is found in the blood, sputum, and urine of the patients. A high affinity recombinant antibody found to interact only with array-printed synthetic carbohydrates containing linear α(1-2)Man linkages, as present in LAM caps, was shown to bind pathogenic mycobacterial species and demonstrated improved sensitivity in the detection of tuberculosis over standard diagnostic methodologies, particularly when urine and serum clinical specimens were tested combinedly (Chan et al., 2015).

On the other hand, immunotherapy using antibodies targeting bacterial surface polysaccharides could be a valuable alternative for fighting infections produced by antibiotic-resistant bacteria, such as carbapenem-resistant *K. pneumoniae*. Two mAbs displaying distinct binding patterns to a microarray containing its CPS repeating unit and fragments thereof were found to be protective against the most virulent clinical strains of this bacterium, promoting their killing and preventing the spread of infection in a murine model (Diago-Navarro et al., 2018). Thus, they can be considered candidates for an antibody-based approach to treat patients infected with carbapenem-resistant *K. pneumoniae*, for which therapeutic options are scarce.

## Bacterial Glycan Arrays for Identification of Ligands for Lectins of the Innate Immune System

**FIGURE 6:**
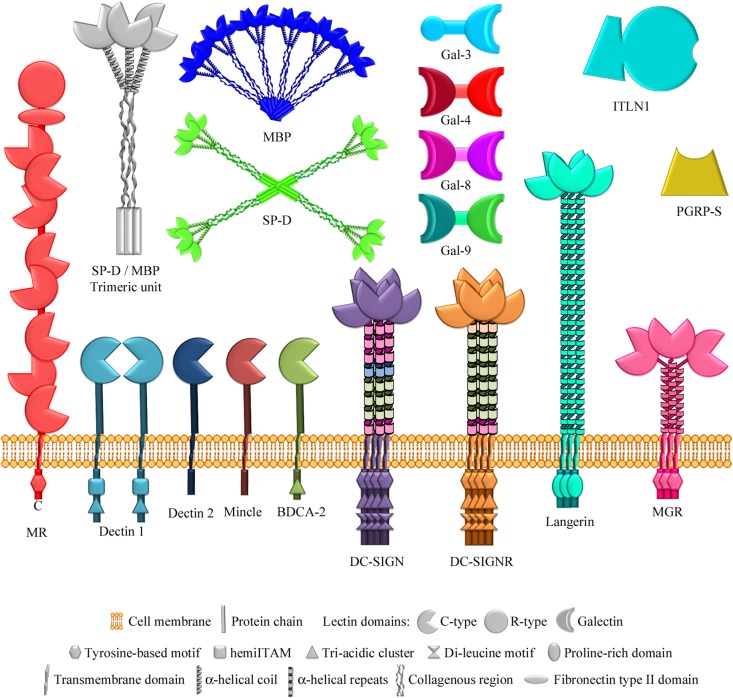
Lectins of the innate immune system examined using bacterial glycan microarrays. The lectins studied comprise several multimodular membrane receptors that contain C-type carbohydrate-recognition domains (Solís et al., 2015) in addition to other non-lectin domains, specified in each case. The mannose receptor also contains an R-type lectin domain. Soluble lectins examined also include two C-type lectins of the collectin family (so called because they contain a collagenous region), which form different multimeric structures based on similar trimeric units. Other soluble lectins studied are different members of the galectin family, belonging to the chimera type (containing one carbohydrate-recognition domain) and tandem-repeat type (containing two different carbohydrate-recognition domains) structural subgroups, intelectin-1 (a member of the X-type lectin family), and a peptidoglycan recognition protein. MR, mannose receptor; BDCA-2, blood dendritic cell antigen 2; DC-SIGN, dendritic cell-specific ICAM-3-grabbing nonintegrin; DC-SIGNR, endothelial cell DC-SIGN homolog; MGR, macrophage galactose receptor; SP-D, surfactant protein D; MBP, serum mannose-binding protein; Gal-3/4/8/9, galectins 3/4/8/9; ITLN1, intelectin-1; PGRP-S, short peptidoglycan recognition protein.

While the antibody-mediated (acquired) immune response requires time to develop after an antigenic challenge, the innate immune response is immediate and it does not require previous exposure to the pathogen, thus being the first line of defense against infection. A variety of lectins that recognize specific glycans on pathogens’ surfaces make an important contribution to innate immune protection. The use of microarrays incorporating bacterial glycan structures has greatly facilitated the identification of ligands and dissection of glycotopes recognized by these lectins (see Figure 6 for schematic representation of lectins of the innate immune system cited in this review).

The value of the approach was demonstrated in a study by Palma et al. (2006) on the assignment of carbohydrate-binding specificity for Dectin-1, the major receptor of the innate immune system on leucocytes against fungal pathogens. The binding of Dectin-1 to a microarray containing 187 neoglycolipids, prepared by reductive amination from selected fractions of *Saccharomyces cerevisiae*, *Alcaligenes faecalis* and *Umbilicaria papulosa* glucan polysaccharides, and from other diverse glycans including many mammalian type glycans, was examined. Remarkably, exclusive binding of Dectin-1 to 10-mer or longer β(1-3)-linked gluco-oligosaccharides, as present in *A. faecalis* glucan curdlan, was detected. This strict requirement of long β(1-3)-linked chains for binding was confirmed in a later study, in which a total of 153 gluco-oligosaccharide neoglycolipids from plant, fungal, and bacterial glucan polysaccharides were prepared by oxime-ligation (Palma et al., 2015). In contrast, the innate immune receptor DC-SIGN (dendritic cell-specific ICAM-3-grabbing nonintegrin) exhibited a broad binding profile, which included recognition of linear β(1-2)-gluco-oligosaccharides derived from the cyclic β(1-2)-glucan of *Brucella abortus* (Palma et al., 2015). Using a focused (1-2)-glucan array, binding of the closely related endothelial cell receptor DC-SIGNR (also named L-SIGN) and of serum mannose-binding protein to linear α and β(1-2)-gluco-oligosaccharides was also observed, although showing distinct lectin-specific binding patterns and differing influence of linkage configuration and chain length (Zhang et al., 2016). Moreover, DC-SIGN was found to recognize intact forms of cyclic *B. abortus* β(1-2)-glucan (Figure 5) printed on microarrays using an appropriate immobilization strategy (Table 2). Of note, linear and circular β(1-2)-linked glucans are produced and secreted by different Proteobacteria and are thought to be involved in biofilm formation, interactions with the host, and modulation of immune cells activities. Overall, these studies evidenced that although these four C-type (Ca^2+^-dependent) lectins of the innate immune system, i.e., Dectin-1, DC-SIGN, DC-SIGNR, and mannose-binding protein, recognize glucans, their fine binding specificities are noticeably different.

Analogous observations were made when the binding patterns of several membrane C-type lectin receptors to an array of mycobacterial glycans were compared. As mentioned above, mycobacteria display unusual surface glycoconjugates. In addition to AM and LAM, they comprise phosphatidyl-myo-inositol mannosides, phenolic glycolipids, glycopeptidolipids, trehalose mycolates, trehalose-containing LOSs, and capsular α-glucans (Figure 1, right part). An array containing 60 chemically synthesized glycans, representing all these classes of mycobacterial structures, was screened with a panel of seven human C-type lectins as well as with bovine mincle (Zheng R. B. et al., 2017), all of them found on the surface of macrophages and/or dendritic cells. No ligands were identified for the macrophage galactose receptor, consistent with its specificity for GalNAc, which was absent from the array. Appropriate ligands were neither present for blood dendritic cell antigen 2 (BDCA-2). Although in this case the primary binding site does bind Man, additional contacts with a Gal residue at a secondary site are known to be required for high-affinity binding. In contrast, DC-SIGN strongly bound to LAM cap structures containing Man residues without a clear preference for particular types of glycans, and it was apparently able to bind internal Man residues. Binding of DC-SIGN to several LAM core structures, possibly inaccessible in the cell wall, and to a phosphatidyl-myo-inositol derivative with terminal Manα(1-2)Man was also detected. Interestingly, a different study revealed binding to array-printed mycobacterial phosphatidylinositol mono- and di-mannosides of the human soluble lectin ZG16p (Hanashima et al., 2015), putting forward a possible involvement of this lectin in the gastrointestinal immune system. The mannose receptor, present on macrophages and sinusoidal endothelial cells, was found to recognize several LAM cap and core structures (Zheng R. B. et al., 2017). However, its binding pattern clearly differed from that of DC-SIGN, as the presence of terminal Man residues was a main factor for recognition. Indeed, the mannose receptor bound to several glycans bearing a single terminal Man, including a phenolic glycolipid. Three other Man-specific lectins, DC-SIGNR, Dectin-2 (from macrophages and dendritic cells), and langerin (present on Langerhans cells), also showed preferential binding to ligands containing exposed Man, although distinctive recognition patterns were visible (Zheng R. B. et al., 2017). For example, langerin bound ligands bearing single terminal Man residues, in addition to more complex LAM structures, whereas Dectin-2 and DC-SIGNR showed a more restricted binding profile, with predominant recognition of Manα(1-2)Man-containing structures. In striking contrast, bovine mincle, found in macrophages and other antigen-presenting cells, bound to a distinct set of mycobacterial glycans containing trehalose (Glcα(1-1)αGlc), independently on variations in substituents, including additions to the 4- or 6-hydroxyl groups of one of the Glc residues. Thus, there was a clear non-overlap between mycobacterial ligands for mincle and for the other Man-specific receptors tested, which also showed distinctive binding preferences.

In a different study, DC-SIGN was also found to interact with the α(1-6)-mannan backbone of lipomannan, another important glycolipid of the *M. tuberculosis* cell wall (Figure 1, right part). Comparison of DC-SIGN binding to array-printed mannans containing 7, 13, and 19 α(1-6)-linked Man units revealed a clear preference for the longer chains (Leelayuwapan et al., 2017), again indicating that this receptor is able to bind internal Man residues. Moreover, binding of DC-SIGN to other microbial glycans was recently observed using a microarray containing 120 synthetic bacterial structures out of over 300 structures (Geissner et al., 2019). In addition to a *M. tuberculosis* AM hexasaccharide displaying terminal Man, DC-SIGN was found to recognize *N*-acetyl-mannosamine (ManNAc)-terminating oligosaccharides based on the CPS of *S. pneumoniae* serotypes 4 and 9. Furthermore, binding to several structures with terminal Hep, based on the LPS inner core of *H. influenzae*, *N. meningitidis, Proteus* sp., and *Yersinia pestis*, was also detected, with α(1-2)- (*H. influenzae*, Figure 5) and α(1-3)- (*N. meningitidis* and *Proteus*) linked Hep being more efficiently bound than α(1-7)-linked Hep (*Y. pestis*). These results further highlighted the plasticity of DC-SIGN’s binding site for accommodating Man-related structures, even bearing substituents at positions 2 (as in ManNAc) or 6 (as in Hep), thereby allowing this receptor to recognize a broad range of microbial ligands.

The ability of human langerin to recognize bacterial glycans different from those displayed by mycobacteria was explored using a microarray containing a collection of 48 bacterial polysaccharides obtained by mild acid hydrolysis of diverse LPSs (O-chain and core) (Feinberg et al., 2011). Langerin bound to *E. coli* and *Shigella boydii* polysaccharides containing Manα(1-2)Man units, indicating that this is an important glycotope for langerin recognition. However, binding to these structures was not detected in a later study using an extended microarray, comprising over 300 bacterial polysaccharides, intact LPSs, and CPSs from a broad range of Gram-negative and -positive bacteria [microbial glycan microarray of the Consortium for Functional Glycomics (CFG)^2^]. Here, only weak binding signals were observed for some non-Man-containing *Shigella* and *Yersinia* antigens, whereas robust binding to yeast mannan was found (Hanske et al., 2017). Unfortunately, as none of these structures were tested in the previous microarray, it is not possible to compare the relative binding intensities obtained in these two studies. Therefore, a further analysis of the binding of human langerin to the spotted *E. coli*, *Shigella*, and *Yersinia* antigens is required. In contrast, despite exhibiting structurally and thermodynamically identical binding to Man and Manα(1-2)Man, murine langerin recognized a broad set of oligosaccharides with highly heterogeneous structures, what could be due to the presence in the murine form of a secondary site, adjacent to the canonic binding site and able to establish interactions with large glycans (Hanske et al., 2017). This interspecies variability could be the result of distinct evolutionary pressures imposed by the different expression patterns of murine and human langerins and their exposure to microbes.

The above-mentioned collections of 48 and 300 bacterial glycans were also used to examine the binding of three members of the galectin family belonging to two different structural subgroups, i.e., galectin 3 (of chimera type), and galectins 4 and 8 (of tandem-repeat-type) (Figure 6). Galectins are a family of lectins widely expressed in epithelial and immune cells and involved, among many other biological phenomena, in inflammation and immunity. In the 48-glycan array, a unique selectivity for the O antigen of *Providencia alcalifaciens* O5 was observed. Importantly, binding of the three galectins to the intact bacterium resulted in loss of viability, demonstrating the utility of the microarray to unveil host−bacteria interactions of functional significance (Stowell et al., 2014). Further analysis of galectin binding to the expanded set of 300 bacterial glycans revealed recognition of a diversity of species presenting mammalian-like carbohydrate determinants, as *K. pneumoniae*, *E. coli*, *P. alcalifaciens*, *Proteus vulgaris*, and *S. pneumoniae* (Stowell et al., 2014). These results demonstrated the ability of galectins to target bacteria displaying self-like antigens. In striking contrast, intelectin-1, a member of the X-type lectin family suspected to be involved in innate immunity, bound in this extended array to ligands containing β-linked galactofuranose, saccharide residues with D-glycerol-1-phosphate substituents, Hep, D-glycero-D-talo-oct-2-ulosonic acid, or Kdo residues, which are widely distributed in bacteria but are not found in mammalian glycans (Wesener et al., 2015). These two studies beautifully illustrate the complementarity in the recognition of self-like and non-self bacterial glycan epitopes by soluble lectins of the innate immune system.

Besides, the binding of tandem-repeat type galectins 4, 8, and 9 to a different microarray incorporating a collection of nearly 150 polysaccharides obtained by mild acid degradation of LPSs from six different bacteria genera (*Escherichia*, *Shigella*, *Salmonella*, *Cronobacter*, *Proteus*, and *Pseudomonas*) was examined (Knirel et al., 2014). Although galectins are characterized by a canonical β-galactoside-binding ability, several β-galactoside-containing polysaccharides with no forbidden substituents at the Gal moieties were not recognized by these galectins. Moreover, binding to non-βGal polysaccharides was detected. Keeping in mind that natural polysaccharides are heterogeneous and may contain minor populations that could account for the observed behavior, this study put forward the binding of galectins to non-canonical determinants.

Surfactant protein D (SP-D) is a different soluble lectin of the innate immune system known to recognize LPSs of several Gram-negative bacteria, triggering agglutination and phagocytosis. SP-D belongs to the C-type collectin family and binds to the LPS inner core Hep constituent. To get insights into the influence of adjacent residues and Hep linkages, the binding of SP-D to a glycan array containing 12 different synthetic inner core structures was examined (Reinhardt et al., 2016). Preferred binding to ligands containing tri-Hep terminal sequences over shorter substructures was observed, the presence of an internal Kdo having no detrimental effect on the recognition. However, replacement of the external Hep moiety by GlcNAc resulted in decreased binding. Moreover, a slight preference for terminal α(1-2)- over α(1-7)-linked Hep was observed. Overall, the results demonstrated SP-D binding to LPS inner core structures present in, e.g., *H. influenzae*, *Enterobacteriaceae*, *Proteus*, or *N. meningitidis*.

Other mammalian effectors of the immune system recognize bacterial cell wall peptidoglycans and activate antimicrobial defense systems, as, e.g., the so called peptidoglycan recognition proteins (PGRPs). However, the recognized motifs are poorly characterized. A series of peptidoglycan fragments consisting of MurNAcβ(1-4)GlcNAc (MG, MurNAc standing for *N*-acetylmuramic acid), (MurNAcβ(1-4)GlcNAc)_2_ (MGMG), or (GlcNAcβ(1-4)MurNAc)_2_ (GMGM), conjugated to di- (L-Ala-D-isoGln), tri- (L-Ala-D-isoGln-L-Lys), or tetrapeptides (L-Ala-D-isoGln-L-Lys-D-Ala), were tested for the binding of human PGRP-S (PGRP short, also known as PGRP 1) (Wang et al., 2016). In accordance with previous data, PGRP-S showed a preference for GMGM conjugates with tri- and tetra-peptides over the dipeptide. In addition, PGRP-S was also found to bind MGMG sequences, again with preference for tri- and tetrapeptide-containing structures. Although this could indicate that peptide length is important for recognition, as interpreted by the authors, the possibility that the Lys residue at position 3 of the tri-/tetra-peptides could be specifically involved in the binding should not be excluded.

In summary, a range of microarrays incorporating diverse bacterial glycans, from large collections to more focused libraries of a specific glycan type or bacterial origin (Table 2), have been selectively used to investigate the binding behavior of different lectins of the innate immune system, unveiling a repertoire of complementary recognition profiles and broad to very strict binding specificities, depending on the particular lectin.

## Bacterial Glycan Arrays for the Study of Ligands Recognized by Bacterial and Phagic Glycan Binding Proteins

Bacteria frequently use surface-exposed lectins to bind to host glycans that serve as docking points for adhesion, and different glycan microarray platforms mainly built with mammalian glycan libraries have been used to get insights into their mode of binding and potential ligands (Flannery et al., 2015; Poole et al., 2018) or to evaluate bacterial adhesion and the efficiency of antiadhesive compounds (Kalograiaki et al., 2018a). In addition, some of these lectins are involved in the formation of bacterial microcolonies and biofilms through binding to glycans present on the surface of neighbor cells or to secreted exopolysaccharides. This is the case for several lectins from *Pseudomonas aeruginosa* and *Burkholderia cepacia* species, two bacteria that can even form mixed biofilms (Elias and Banin, 2012). The binding specificity of *P. aeruginosa* lectins PA-IL and PA-IIL (also referred to as LecA and LecB) and of *B. cenocepacia* lectins A and C (designated BC2L-A and BC2L-C) was investigated using glycan arrays from the Consortium for Functional Glycomics. Despite PA-IIL and BC2L-A are closely related Man-binding lectins, PA-IIL was found to show preference for fucosylated oligosaccharides (Marotte et al., 2007), while BC2L-A only bound to oligomannose glycans (Lameignere et al., 2008). Interestingly, screening of the two separate carbohydrate-recognition domains of BC2L-C revealed binding of the N-terminal domain to fucosylated oligosaccharides and of the C-domain to Man-terminated glycans (Sulak et al., 2011), thus combining in one single lectin binding properties similar to those of PA-IIL and BC2L-A. In contrast, PA-IL showed a high specificity for terminal α-Gal, with preference for Galα(1-4)Gal-terminating structures (Blanchard et al., 2008). Although the arrays tested were based on mammalian glycan structures, binding of these lectins to similar sugar epitopes in bacterial glycans, as, e.g., in the Gal- and Man-rich exopolysaccharide of *P. aeruginosa* (Psl), could be extrapolated. In addition, PA-IIL, BC2L-A, and BC2L-C could recognize Hep residues of LPSs, as described above for the Man-specific innate immune lectins DC-SIGN and SP-D. Indeed, binding of BC2L-A to Hep mono- and disaccharides, and Hep-containing oligo- and polysaccharides was later confirmed using a combination of NMR spectroscopy, X-ray crystallography, and calorimetry techniques (Marchetti et al., 2012). Moreover, binding of BC2L-A and the C-domain of BC2L-C to microarray-printed Man or Hep terminating bacterial glycans has very recently reported, this study unveiling a preference of both lectins for α(1-6)- over α(1-2)-linked Hep (Geissner et al., 2019). All in all, a study of the binding patterns to appropriate bacterial glycan arrays would certainly be of great help to identify the precise structures recognized by the bacterial lectins. This in turn will facilitate the design of new antibacterial agents able to interfere with microcolony and biofilm formation.

Many bacterial glycoside hydrolases contain non-catalytic CBMs that target the enzyme to specific regions on its substrate and promote hydrolysis. A finely tuned selectivity in carbohydrate recognition was demonstrated in a study on the binding of six bacterial glucan-binding CBMs to the collection of 153 gluco-oligosaccharide neoglycolipids described in the preceding section (Palma et al., 2015). The analysis confirmed known binding preferences, with distinct CBM-specific profiles and differing influence of oligosaccharide sequence and length, and also revealed novel binding specificities including recognition of bacterial glycans. For example, CBM41 from *Thermotoga maritima* pullulanase showed the predicted binding to α(1-4)-gluco-oligosaccharides, and the presence of α(1-6)-linked Glc was found to be better tolerated at an internal position than at the non-reducing end. Binding of CBM11 from *Clostridium thermocellum* endoglucanase was in agreement with its selectivity toward Glcβ(1-4)Glcβ(1-4)Glcβ(1-3)Glc, although only heptasaccharide and longer chains were bound, suggesting that chain length is important for recognition. In the case of CBM4-2 from *T. maritima* laminarinase, which showed preferential binding to linear β(1-3)-glucans, a minimum chain length of four units was required, while for two CBMs from *Cellvibrio mixtus* cellulase (CBM32-2) and *Bacillus halodurans* laminarinase (CBM6), showing prominent binding to linear β(1-2)- and β(1-3)-, as well as branched β(1-3/6)-oligosaccharides, binding to di- and tri-saccharide structures could be detected. Finally, CBM6-2 from *C. mixtus* endoglucanase 5A, which contains two binding clefts with different specificity, exhibited the broadest recognition profile, including binding to linear β(1-2)-gluco-oligosaccharides derived from the cyclic β(1-2)-glucan of *B. abortus*, not reported previously for this CBM. This study demonstrated the effectiveness of the microarray as a tool for probing recognition of short as well as long bacterial glucans.

A different module present in bacterial peptidoglycan hydrolases is the Lysin Motif (LysM) domain. This is a widespread domain, found in proteins from viruses to mammals, that recognizes polysaccharides containing GlcNAc residues, as present in the bacterial cell wall peptidoglycan or in chitin, the main constituent of fungal cell walls. Furthermore, plant receptors containing LysM domains recognize lipochitin oligosaccharides that are synthesized by nitrogen fixing bacteria to be used as signaling molecules (Nod factors) in legume–rhizobium symbiosis. A microarray containing a series of natural and synthetic Nod factors, chitin oligosaccharides, and peptidoglycan-related compounds was developed to investigate interactions involving LysM domain-containing proteins (Maolanon et al., 2014). Analysis of the binding to the array of P60, an autolysin of *Listeria monocytogenes* that hydrolyzes the cell wall peptidoglycan and is essential for bacterial virulence, revealed recognition of chitin oligosaccharides of ≥ 5 GlcNAc units and selective binding to some Nod factors, in particular those containing a C18:1 lipid chain. Interestingly, a chemically synthesized LysM domain of the Nod factor receptor 5 from the legume *Lotus japonicas* was also found to show preference for Nod factors with C18:1 lipid chains (Sorensen et al., 2014). Thus, the bacterial and plant LysM domains appeared to exhibit a similar dependence on the lipid structure, hinting at a possible role of the lipid moiety in the binding of LysM domains to Nod factors.

Bacteriophages also exploit recognition of specific bacterial glycan motifs for invading their hosts. An example is the lytic phage NCTC 12673, which recognizes the capsular polysaccharide of only a limited number of *C. jejuni* isolates, thereby conferring the phage its high host specificity. In addition, NCTC 12673 contains a putative binding protein, Gp047, with a much broader host-binding range than the phage, being capable of binding to multiple *C. jejuni* and *Campylobacter coli* strains. This protein was found to recognize flagellin decorated with acetamidino-modified pseudaminic acid. However, it did not bind any of the structures printed in the 48-glycan array mentioned before, not even to glycans of *P. aeruginosa* containing acetimidoyl groups, thus evidencing the specific requirement for recognition of the acetamidino modification on pseudaminic acid (Javed et al., 2015). The authors proposed that Gp047 could be released from phage-infected bacteria and bind to flagella of neighboring *C. jejuni* cells, thereby reducing their motility and assisting in the next round of infection.

## Bacteria Microarrays for Examining Bacterial Surface Glycans and Their Recognition by Glycan-Binding Proteins

The potential of microarrays incorporating natural or synthetic bacterial glycan structures for exploring the recognition of bacteria by carbohydrate-binding proteins is evidently limited by the library of probes included in the array. If the particular carbohydrate structure recognized by the protein is not present in the array, the analysis may be misinterpreted as a lack of binding to a given bacterium. On the other hand, the presentation of the glycan probes in the array in an accessible and clustered (high density) form may substantially differ from their natural arrangement and accessibility on the bacterial surface. Therefore, the possibility that the results do not correlate with the real bacteria−receptor interplay does exist and poses a challenge to the design of microarray-based strategies. Moreover, synergistic operative interactions of the glycan-binding proteins with other cell surface molecules cannot be evaluated. Therefore, besides assessing recognition of isolated bacterial components by glycan-binding proteins for identification of ligand candidates, analysis of binding to bacterial supramolecular structures and entire cells is needed.

An example is the study of the recognition of the bacterial peptidoglycan by the cell wall-binding domain of the endolysin Cpl-7, which is encoded by the pneumococcal Cp-7 bacteriophage (Bustamante et al., 2017). Cpl-7 is composed of a catalytic domain with muramidase activity and a cell wall-binding domain (C-Cpl-7) made up of three CW_7 repeats. Although these repeats have only been characterized in two other endolysins, they are present in many putative cell wall hydrolase sequences, suggesting that they target a conserved element of the bacterial cell wall. Inspection of the mode of binding of peptidoglycan fragments to C-Cpl-7 using a combination of X-ray crystallography, saturation transfer difference NMR spectroscopy (STD-NMR), and docking studies, unveiled GlcNAcβ(1-4)MurNAc-L-Ala-D-isoGln as the minimal recognized fragment and the involvement, among other contacts, of a fully conserved arginine residue in hydrogen bonding with the GlcNAc moiety. Binding assays of C-Cpl-7 to cell wall fragments from the laboratory *S*. *pneumoniae* strain R6, grown in choline- or ethanolamine-containing media (i.e., with choline- or ethanolamine-containing TAs), printed on nitrocellulose-coated glass slides, confirmed recognition of the pneumococcal cell wall independently of the choline or ethanolamine decoration of TAs (Bustamante et al., 2017). Moreover, upon substitution in the three repeats of the mentioned arginine residue by alanine, what did not alter the protein structure, a decrease of around 50% in the intensity of the binding signals was observed. These results suggested that the mode of binding to the complex peptidoglycan layer is likely to be analogous to that defined for the small glycopeptide studied.

As endolysins break down the cell wall from the inside of bacteria to release the phage progeny, the use of cell wall fragments to investigate endolysins’ recognition of the bacterial peptidoglycan (or of TAs) is indicated. However, the interaction of glycan-binding proteins with bacterial surface glycans should be better explored using microarray-printed entire cells. This approach was used to detect antibodies against cell surface antigens (Thirumalapura et al., 2006). Microarrays containing inactivated *E. coli*, *F. tularensis*, *K. pneumoniae*, *S.* Typhimurium, *E. faecalis*, *S. aureus*, *S. epidermidis*, *Streptococcus pyogenes*, and *L. monocytogenes* cells were tested for the binding of mAbs against *F. tularensis* O-antigen, *Salmonella* O-antigen (B group), *S. aureus* peptidoglycan, and *L. monocytogenes*. Specific recognition of the respective bacteria was demonstrated with no meaningful cross-reactions. To assess the utility of the approach for antibody detection in clinical samples, seven canine serum samples from clinical cases of tularemia and positive for *F. tularensis* antibodies, along with six canine serum samples negative for *F. tularensis* antibodies, were comparatively tested. Significantly higher levels of anti-*F. tularensis* antibodies were detected in tularemia positive samples compared to negative samples (Thirumalapura et al., 2006). In addition, variable levels of antibodies against the other bacteria were also observed, showing that simultaneous detection of different anti-bacteria antibodies was possible.

More recently, bacteria microarrays have proved to be useful for exploring the presence of carbohydrate structures on bacterial surfaces (Campanero-Rhodes et al., 2015; Kalograiaki et al., 2016, 2018b). *K. pneumoniae* O1:K2 strain 52145, a clinically relevant serotype, was first used as model bacterium (Campanero-Rhodes et al., 2015). This strain displays a Gal-containing O-chain (Figure 5) and a CPS built by a branched Glc/Man-based tetrasaccharide repeating unit, which are glycan structures commonly found in isolates from *K. pneumoniae*-infected individuals. By testing the binding of a panel of 10 lectins of known binding specificities to array-printed *K. pneumoniae* 52145 cells, the accessibility for lectin recognition of Gal- and Man/Glc-containing structures on the bacterial surface was confirmed. A series of isogenic mutants lacking the capsule, the LPS O-chain and/or the major outer membrane protein OmpA, printed in parallel, helped to dissect the specific structures recognized by those lectins giving meaningful binding signals toward the wild type strain. A strong preference of the Gal-specific lectins RCA and PNA (Table 3) for non-capsulated and O-chain-containing strains was evident, pointing to the O-chain as the primary recognized epitope. In the case of the Man/Glc-specific lectin ConA, the results indicated that the CPS was not the main recognized structure, as there was no preference for capsulated over non-capsulated strains. Importantly, for all the lectins the binding signals were reduced down to background levels when the binding assays were carried out in the presence of their specific haptens, thereby proving that lectin binding was carbohydrate mediated. Therefore, other glycan structures different from the CPS were apparently recognized by ConA.

The efficiency of bacteria microarrays for exploring the presence of surface glycans of bacteria not presenting CPS and O-antigen-containing LPS was next demonstrated using non-typeable *H. influenzae* (NTHi) as model (Kalograiaki et al., 2016). Binding assays to microarray-printed NTHi strain 375 (hereafter referred to as NTHi375) with a panel of 19 lectins revealed positive and hapten-inhibitable signals for Gal-, Glc-, and sialic acid-specific lectins, indicating the presence on the bacterial surface of carbohydrate structures specifically recognized by the lectins. Analysis of lectin binding to a set of isogenic mutant strains expressing sequentially truncated LOS supported the notion that the LOS could be a target for most lectins. Interestingly, LOS truncation had disparate consequences on the binding of the Gal-specific lectins VAA and RCA (Table 3). In particular, the absence of the Galα(1-4)Galβ epitope from the chain extension linked to the distal manno-heptose of the Hep trisaccharide inner core (Figure 5) resulted in decreased binding of VAA but had no significant effect on the binding of RCA, suggesting that RCA might not bind this LOS. Indeed, a follow up study using microarrays containing the purified LOS showed only marginal binding of RCA as opposed to strong binding of VAA, which was drastically reduced for a truncated LOS lacking Galα(1-4)Galβ (Kalograiaki et al., 2018c). In striking contrast, RCA bound strongly to the microarray-printed LOS from the capsule-deficient *H. influenzae* laboratory strain RdKW20, whose major glycoform displays terminal Galβ(1-4)Glc at the distal Hep extension, while, although ∼19% of this LOS bears terminal Galα(1-4)Galβ, VAA bound only weakly. In-depth analysis of the LOS epitopes recognized by RCA and VAA in each case, using STD-NMR experiments assisted by molecular dynamics simulations, revealed that RCA bound the RdKW20 LOS glycoform displaying terminal Galβ(1-4)Glcβ, whereas VAA recognized the Galα(1-4)Galβ(1-4)Glcβ epitope (Figure 5) in NTHi375 LOS but not in RdKW20 LOS, what could be due to different conformational preferences of the branch and ensuing presentation of the epitope. Binding assays to wild type and selected mutant/transformed whole bacterial cells ran in parallel revealed that, besides the LOS, other carbohydrate structures on the bacterial surface serve as lectin ligands, and highlighted the impact of the specific display of cell surface components on lectin binding, stressing the importance of examining binding to entire bacterial cells (Kalograiaki et al., 2018c).

Having proved the utility of bacteria microarrays for exploring the presence of carbohydrate structures on the surface of the model NTHi375 strain, the glycosignatures of five other NTHi clinical isolates from otitis media and COPD (chronic obstructive pulmonary disease) patients and from pediatric healthy carriers were examined (Kalograiaki et al., 2016). Different lectin-binding fingerprints were observed, consistent with the known inter-strain heterogeneity of *H. influenzae* LOS, which is linked to variable outcomes with the host, i.e., colonization, persistence, or acute infection. Again, RCA and VAA exhibited different binding patterns, supporting that these two lectins recognize different ligands on the NTHi surface. At any rate, the results evidenced the availability on the bacterial surface of Man/Glc, Gal, and sialic acid residues that could be recognized by lectins of the innate immune system with the appropriate carbohydrate-binding specificity. Indeed, analysis of the binding of SP-D, galectin-8, and Siglec-14, which exhibit Man/Glc, Gal, and sialic acid binding specificities, respectively, to the array-printed NTHi clinical isolates revealed lectin- and strain-specific recognition (Kalograiaki et al., 2016), providing the first experimental evidence for direct binding of SP-D to NTHi and also demonstrating binding of galectin-8 and Siglec-14 to NTHi strains other than NTHi2019, previously reported (Angata et al., 2013; Stowell et al., 2014). Overall, the microarray analysis afforded information on the glycosignatures of the tested bacteria and detected recognition by host receptors, providing semiquantitative data on binding avidity.

## Other Applications of Lectin, Antibody, and Bacterial Glycan Microarrays

The main focus of this review was the description of microarray strategies for exploring the presence of glycans on bacterial surfaces and their interactions with a diversity of glycan-binding proteins. Still, other interesting microarray approaches that could be of value to the microbiologist community deserve to be mentioned.

A first example is the use of a sandwiched microarray platform for testing the efficiency of antibiotics on *S. aureus* growth (Liu et al., 2016). A lectin-hydrogel microarray was employed for capturing bacteria, and a matching drug-laden polyacrylamide microarray was then used for building microchambers between the two microarrays, in which live bacteria were co-cultured with antibiotics. Minimum inhibitory concentrations obtained in this way for four well-known antibiotics were in agreement with reported values.

Antibody microarrays have been used for detecting bacteria in a diversity of samples, covering from water to rocks. For example, *E. coli* O157:H7, *S.* Typhimurium, and *Legionella pneumophila*, which are responsible for water-borne infections, were detected in water using a flow-through chemiluminescence microarray approach (Wolter et al., 2008; Karsunke et al., 2009). A method for fast detection of clinically relevant levels of *S. Enteritidis* in blood, based on monitoring of bacterial growth by SPRi, has also been reported (Templier et al., 2017). The method was claimed to be of value for detecting bloodstream bacterial infections (bacteremia) using blood volumes similar to those used in standard analyses. Moreover, microarrays containing collections of up to 300 antibodies have been used for environmental monitoring of bacteria, in, e.g., aquatic ecosystems (Blanco et al., 2015), or even detection of “signs of life” in solid samples, including sediments, rocks, and subsoil samples, especially those from extreme environments such as the hypersaline Atacama subsurface (Parro et al., 2011) or the permafrost from the antarctical Deception Island (Blanco et al., 2012). Although most of the antibodies used in these studies did not explicitly target bacterial glycan structures, as they were raised against whole bacterial cells, in some cases detection of bacterial exopolysaccharides, LTAs, and peptidoglycans was demonstrated (Rivas et al., 2008; Parro et al., 2011; Blanco et al., 2012; Puente-Sánchez et al., 2014). Of note, the successful results obtained with these arrays supported their utility for planetary exploration, as, e.g., the search for life in Mars.

Regarding carbohydrate microarrays, a singular approach involving binding assays of purified bacterial glycans to microarray-printed host glycans was used to examine host–bacteria glycan–glycan interactions. Numerous interactions of LOS/LPSs isolated from *C. jejuni*, *H. influenzae*, *S.* Typhimurium, and *Shigella flexneri* with the printed probes were detected (Day et al., 2015). Moreover, cell assays demonstrated that adherence of bacteria to host cells could be inhibited with either host or bacterial glycans, indicating that the observed glycan−glycan interactions could importantly contribute to the binding of bacteria to host cells. As mentioned before, the potential of carbohydrate microarrays for exploring recognition events is limited by the library of probes used for building the arrays, what is frequently restricted by the laborious procedures required for obtaining well-defined glycan structures. Engineered phages displaying specific oligosaccharides, named “glycophages,” have been put forward as an alternative (or complement) to the commonly used glycan libraries of natural and/or synthetic origin (Çelik et al., 2015). In particular, microarray-printed phages displaying the *P. aeruginosa* O11 O-antigen were successfully recognized by serum antibodies against this O-antigen, which did not bind to other glycophages in the array displaying O-antigen polysaccharides from *C. jejuni, Campylobacter lari*, *E. coli* O78, *E. coli* O148, *F. tularensis*, or *Shigella dysenteriae*, demonstrating the applicability of the approach. A key advantage of glycophages is that they can be produced in bacteria in large quantities and isolated easily from bacterial supernatants.

## Concluding Remarks

As illustrated in this review, the number and diversity of applications of the microarray technology grow continuously, offering novel and complementary high-throughput tools for bacteria-related studies in multiple areas, from basic science to the clinical or food safety fields and even environmental exploration. Still, several advances are required to maximize the potential of this technology. For example, the binding specificity of microarray-printed lectins has been typically investigated using eukaryotic glycans as ligand candidates. Therefore, their ability to recognize sugar residues and structures exclusively found in bacteria is not known in most cases and should be thoroughly examined. Production of recombinant lectins with engineered binding specificities would definitely facilitate a wider and more selective coverage of bacterial glycans. Regarding antibody microarrays, the quality and cross-reactivity of anti-carbohydrate antibodies are important issues to tackle. In addition, increased sensitivity is required to enable their use as routine tool for detection of contaminating bacteria in real-world samples. Expansion of bacterial glycan libraries through improved methods for isolation and structural characterization or chemical/enzymatic synthesis or by exploiting novel alternatives, as the above-mentioned “glycophages,” would enhance the potential of carbohydrate microarrays for exploring recognition events. Common to all types of microarrays is the need of better tools to analyze data and to establish functional correlations, e.g., between bacterial glycosignatures and virulence. These and other developments leading to a greater simplicity and accessibility to this technique will surely broaden its applications in bacterial glycobiology and related areas.

## Author Contributions

MC-R reviewed the literature, prepared the figures and tables, and edited the manuscript. AP and MM contributed to literature search and edited the manuscript. DS conceived the review, contributed to literature search, and wrote the manuscript.

## Conflict of Interest

The authors declare that the research was conducted in the absence of any commercial or financial relationships that could be construed as a potential conflict of interest.

## Footnotes

1 https://www.cdc.gov/ecoli/2019/o103-04-19/index.html

2 http://www.functionalglycomics.org

## References

[B1] Adibekian A. Stallforth P. Hecht M. L. Werz D. B. Gagneux P. Seeberger P. H. (2011). Comparative bioinformatics analysis of the mammalian and bacterial glycomes. *Chem. Sci.* 2 337–344. 10.1093/glycob/cwu092 25190359

[B2] Angata T. Ishii T. Motegi T. Oka R. Taylor R. E. Soto P. C. (2013). Loss of Siglec-14 reduces the risk of chronic obstructive pulmonary disease exacerbation. *Cell. Mol. Life Sci.* 70 3199–3210. 10.1007/s00018-013-1311-7 23519826 PMC3718857

[B3] Angenendt P. (2005). Progress in protein and antibody microarray technology. *Drug Discov. Today* 10 503–511. 10.1016/S1359-6446(05)03392-1 15809196

[B4] Blanchard B. Nurisso A. Hollville E. Tetaud C. Wiels J. Pokorna M. (2008). Structural basis of the preferential binding for globo-series glycosphingolipids displayed by *Pseudomonas aeruginosa* lectin I. *J. Mol. Biol.* 383 837–853. 10.1016/j.jmb.2008.08.028 18762193

[B5] Blanco Y. Prieto-Ballesteros O. Gómez M. J. Moreno-Paz M. García-Villadangos M. Rodríguez-Manfredi J. A. (2012). Prokaryotic communities and operating metabolisms in the surface and the permafrost of Deception Island (Antarctica). *Environ. Microbiol.* 14 2495–2510. 10.1111/j.1462-2920.2012.02767.x 22564293

[B6] Blanco Y. Quesada A. Gallardo-Carreño I. Aguirre J. Parro V. (2015). CYANOCHIP: an antibody microarray for high-taxonomical-resolution cyanobacterial monitoring. *Environ. Sci. Technol.* 49 1611–1620. 10.1021/es5051106 25565212

[B7] Blixt O. Head S. Mondala T. Scanlan C. Huflejt M. E. Alvarez R. (2004). Printed covalent glycan array for ligand profiling of diverse glycan binding proteins. *Proc. Natl. Acad. Sci. U.S.A.* 101 17033–17038. 10.1073/pnas.0407902101 15563589 PMC534418

[B8] Blixt O. Hoffmann J. Svenson S. Norberg T. (2008). Pathogen specific carbohydrate antigen microarrays: a chip for detection of *Salmonella* O-antigen specific antibodies. *Glycoconj J.* 25 27–36. 10.1007/s10719-007-9045-0 17558551

[B9] Blumenschein T. M. A. Friedrich N. Childs R. A. Saouros S. Carpenter E. P. Campanero-Rhodes M. A. (2007). Atomic resolution insight into host cell recognition by *Toxoplasma gondii*. *Embo. J.* 26 2808–2820. 10.1038/sj.emboj.7601704 17491595 PMC1888667

[B10] Bouguelia S. Roupioz Y. Slimani S. Mondani L. Casabona M. G. Durmort C. (2013). On-chip microbial culture for the specific detection of very low levels of bacteria. *Lab Chip* 13 4024–4032. 10.1039/c3lc50473e 23912527

[B11] Broecker F. Hanske J. Martin C. E. Baek J. Y. Wahlbrink A. Wojcik F. (2016a). Multivalent display of minimal *Clostridium difficile* glycan epitopes mimics antigenic properties of larger glycans. *Nat. Commun.* 7:11224. 10.1038/ncomms11224 27091615 PMC4838876

[B12] Broecker F. Martin C. E. Wegner E. Mattner J. Baek J. Y. Pereira C. L. (2016b). Synthetic lipoteichoic acid glycans are potential vaccine candidates to protect from *Clostridium difficile* Infections. *Cell Chem. Biol.* 23 1014–1022. 10.1016/j.chembiol.2016.07.009 27524293

[B13] Bustamante N. Iglesias-Bexiga M. Bernardo-García N. Silva-Martín N. García G. Campanero-Rhodes M. A. (2017). Deciphering how Cpl-7 cell wall-binding repeats recognize the bacterial peptidoglycan. *Sci. Rep.* 7:16494. 10.1038/s41598-017-16392-4 29184076 PMC5705596

[B14] Cai H. Y. Lu L. Muckle C. A. Prescott J. F. Chen S. (2005). Development of a novel protein microarray method for serotyping *Salmonella enterica* strains. *J. Clin. Microbiol.* 43 3427–3430. 10.1128/Jcm.43.7.3427-3430.2005 16000469 PMC1169117

[B15] Campanero-Rhodes M. A. Childs R. A. Kiso M. Komba S. Le Narvor C. Warren J. (2006). Carbohydrate microarrays reveal sulphation as a modulator of siglec binding. *Biochem. Biophys. Res. Commun.* 344 1141–1146. 10.1016/j.bbrc.2006.03.223 16647038

[B16] Campanero-Rhodes M. A. Llobet E. Bengoechea J. A. Solís D. (2015). Bacteria microarrays as sensitive tools for exploring pathogen surface epitopes and recognition by host receptors. *Rsc Adv.* 5 7173–7181. 10.1039/c4ra14570d

[B17] Campanero-Rhodes M. A. Smith A. Chai W. G. Sonnino S. Mauri L. Childs R. A. (2007). N-glycolyl GM1 ganglioside as a receptor for simian virus 40. *J. Virol.* 81 12846–12858. 10.1128/jvi.01311-07 17855525 PMC2169104

[B18] Casals C. Campanero-Rhodes M. A. García-Fojeda B. Solís D. (2018). The role of collectins and galectins in lung innate immune defense. *Front. Immunol.* 9:1998. 10.3389/fimmu.2018.01998 30233589 PMC6131309

[B19] Çelik E. Ollis A. A. Lasanajak Y. Fisher A. C. Gür G. Smith D. F. (2015). Glycoarrays with engineered phages displaying structurally diverse oligosaccharides enable high-throughput detection of glycan-protein interactions. *Biotechnol. J.* 10 199–209. 10.1002/biot.201400354 25263089 PMC4314398

[B20] Chan C. E. Götze S. Seah G. T. Seeberger P. H. Tukvadze N. Wenk M. R. (2015). The diagnostic targeting of a carbohydrate virulence factor from *M.Tuberculosis*. *Sci. Rep.* 5:10281. 10.1038/srep10281 25975873 PMC4432570

[B21] Chen T. Blanc C. Eder A. Z. Prados-Rosales R. Souza A. C. Kim R. S. (2016). Association of human antibodies to arabinomannan with enhanced mycobacterial opsonophagocytosis and intracellular growth reduction. *J. Infect. Dis.* 214 300–310. 10.1093/infdis/jiw141 27056953 PMC4918826

[B22] Day C. J. Tran E. N. Semchenko E. A. Tram G. Hartley-Tassell L. E. Ng P. S. K. (2015). Glycan:glycan interactions: high affinity biomolecular interactions that can mediate binding of pathogenic bacteria to host cells. *Proc. Natl. Acad. Sci. U.S.A.* 112 E7266–E7275. 10.1073/pnas.1421082112 26676578 PMC4702957

[B23] Diago-Navarro E. Motley M. P. Ruiz-Pérez G. Yu W. Austin J. Seco B. M. S. (2018). Novel, broadly reactive anticapsular antibodies against carbapenem-resistant *Klebsiella pneumoniae* protect from infection. *mBio* 9:e91-18. 10.1128/mBio.00091-18 29615497 PMC5885035

[B24] Elias S. Banin E. (2012). Multi-species biofilms: living with friendly neighbors. *Fems Microbiol. Rev.* 36 990–1004. 10.1111/j.1574-6976.2012.00325.x 22229800

[B25] Emmadi M. Khan N. Lykke L. Reppe K. Parameswarappa S. G. Lisboa M. P. (2017). A *Streptococcus pneumoniae* Type 2 oligosaccharide glycoconjugate elicits opsonic antibodies and is protective in an animal model of invasive Pneumococcal disease. *J. Am. Chem. Soc.* 139 14783–14791. 10.1021/jacs.7b07836 28945368

[B26] Feinberg H. Taylor M. E. Razi N. McBride R. Knirel Y. A. Graham S. A. (2011). Structural basis for langerin recognition of diverse pathogen and mammalian glycans through a single binding site. *J. Mol. Biol.* 405 1027–1039. 10.1016/j.jmb.2010.11.039 21112338 PMC3065333

[B27] Flannery A. Gerlach J. Q. Joshi L. Kilcoyne M. (2015). Assessing bacterial interactions using carbohydrate-based microarrays. *Microarrays* 4 690–713. 10.3390/microarrays4040690 27600247 PMC4996414

[B28] Fukui S. Feizi T. Galustian C. Lawson A. M. Chai W. (2002). Oligosaccharide microarrays for high-throughput detection and specificity assignments of carbohydrate-protein interactions. *Nat. Biotechnol.* 20 1011–1017. 10.1038/nbt735 12219077

[B29] Ganeshapillai J. Vinogradov E. Rousseau J. Weese J. S. Monteiro M. A. (2008). *Clostridium difficile* cell-surface polysaccharides composed of pentaglycosyl and hexaglycosyl phosphate repeating units. *Carbohydrate Res.* 343 703–710. 10.1016/j.carres.2008.01.002 18237724

[B30] Gao J. Liu D. Wang Z. (2010). Screening lectin-binding specificity of bacterium by lectin microarray with gold nanoparticle probes. *Anal. Chem.* 82 9240–9247. 10.1021/ac1022309 20973590

[B31] Gehring A. G. Albin D. M. Bhunia A. K. Reed S. A. Tu S. I. Uknalis J. (2006). Antibody microarray detection of *Escherichia coli* O157 : H7: quantification, assay limitations, and capture efficiency. *Anal. Chem.* 78 6601–6607. 10.1021/ac0608467 16970339

[B32] Geissner A. Pereira C. L. Leddermann M. Anish C. Seeberger P. H. (2016). Deciphering antigenic determinants of *Streptococcus pneumoniae* Serotype 4 capsular polysaccharide using synthetic oligosaccharides. *ACS Chem. Biol.* 11 335–344. 10.1021/acschembio.5b00768 26674834

[B33] Geissner A. Reinhardt A. Rademacher C. Johannssen T. Monteiro J. Lepenies B. (2019). Microbe-focused glycan array screening platform. *Proc. Natl. Acad. Sci. U.S.A.* 116 1958–1967. 10.1073/pnas.1800853116 30670663 PMC6369816

[B34] Hanashima S. Gotze S. Liu Y. Ikeda A. Kojima-Aikawa K. Taniguchi N. (2015). Defining the Interaction of Human Soluble Lectin ZG16p and Mycobacterial Phosphatidylinositol Mannosides. *Chembiochem* 16 1502–1511. 10.1002/cbic.201500103 25919894 PMC5896728

[B35] Hanske J. Schulze J. Aretz J. McBride R. Loll B. Schmidt H. (2017). Bacterial polysaccharide specificity of the pattern recognition receptor langerin is highly species-dependent. *J. Biol. Chem.* 292 862–871. 10.1074/jbc.M116.751750 27903635 PMC5247659

[B36] Hegde N. V. Praul C. Gehring A. Fratamico P. DebRoy C. (2013). Rapid O serogroup identification of the six clinically relevant Shiga toxin-producing *Escherichia coli* by antibody microarray. *J. Microbiol. Methods* 93 273–276. 10.1016/j.mimet.2013.03.024 23570904

[B37] Herget S. Toukach P. V. Ranzinger R. Hull W. E. Knirel Y. A. von der Lieth C. W. (2008). Statistical analysis of the bacterial carbohydrate structure data base (BCSDB): characteristics and diversity of bacterial carbohydrates in comparison with mammalian glycans. *BMC Struct. Biol.* 8:35. 10.1186/1472-6807-8-35 18694500 PMC2543016

[B38] Hsu K. L. Pilobello K. T. Mahal L. K. (2006). Analyzing the dynamic bacterial glycome with a lectin microarray approach. *Nat. Chem. Biol.* 2 153–157. 10.1038/nchembio767 16462751

[B39] Jankute M. Cox J. A. Harrison J. Besra G. S. (2015). Assembly of the mycobacterial cell wall. *Annu. Rev. Microbiol.* 69 405–423. 10.1146/annurev-micro-091014-104121 26488279

[B40] Javed M. A. van Alphen L. B. Sacher J. Ding W. Kelly J. Nargang C. (2015). A receptor-binding protein of *Campylobacter jejuni* bacteriophage NCTC 12673 recognizes flagellin glycosylated with acetamidino-modified pseudaminic acid. *Mol. Microbiol.* 95 101–115. 10.1111/mmi.12849 25354466

[B41] Kalograiaki I. Abellán-Flos M. Fernández L. A. Menéndez M. Vincent S. P. Solís D. (2018a). Direct evaluation of live uropathogenic *Escherichia coli* adhesion and efficiency of antiadhesive compounds using a simple microarray approach. *Anal. Chem.* 90 12314–12321. 10.1021/acs.analchem.8b04235 30284810

[B42] Kalograiaki I. Campanero-Rhodes M. A. Proverbio D. Euba B. Garmendia J. Aastrup T. (2018b). Bacterial surface glycans: microarray and QCM strategies for glycophenotyping and exploration of recognition by host receptors. *Methods Enzymol.* 598 37–70. 10.1016/bs.mie.2017.06.011 29306443

[B43] Kalograiaki I. Euba B. Fernández-Alonso M. D. C. Proverbio D. St Geme J. W. III Aastrup T. (2018c). Differential recognition of *Haemophilus influenzae* whole bacterial cells and isolated lipooligosaccharides by galactose-specific lectins. *Sci. Rep.* 8:16292. 10.1038/s41598-018-34383-x 30389954 PMC6215012

[B44] Kalograiaki I. Euba B. Proverbio D. Campanero-Rhodes M. A. Aastrup T. Garmendia J. (2016). Combined bacteria microarray and quartz crystal microbalance approach for exploring glycosignatures of nontypeable *Haemophilus influenzae* and recognition by host lectins. *Anal. Chem.* 88 5950–5957. 10.1021/acs.analchem.6b00905 27176788

[B45] Karsunke X. Y. Niessner R. Seidel M. (2009). Development of a multichannel flow-through chemiluminescence microarray chip for parallel calibration and detection of pathogenic bacteria. *Anal. Bioanal. Chem.* 395 1623–1630. 10.1007/s00216-009-2905-7 19575190

[B46] Kilcoyne M. Twomey M. E. Gerlach J. Q. Kane M. Moran A. P. Joshi L. (2014). *Campylobacter jejuni* strain discrimination and temperature-dependent glycome expression profiling by lectin microarray. *Carbohydr Res.* 389 123–133. 10.1016/j.carres.2014.02.005 24680511

[B47] Knirel Y. A. Gabius H. J. Blixt O. Rapoport E. M. Khasbiullina N. R. Shilova N. V. (2014). Human tandem-repeat-type galectins bind bacterial non-betaGal polysaccharides. *Glycoconj J.* 31 7–12. 10.1007/s10719-013-9497-3 24065176

[B48] Lameignere E. Malinovska L. Slavikova M. Duchaud E. Mitchell E. P. Varrot A. (2008). Structural basis for mannose recognition by a lectin from opportunistic bacteria *Burkholderia cenocepacia*. *Biochem. J.* 411 307–318. 10.1042/bj20071276 18215132

[B49] Leelayuwapan H. Kangwanrangsan N. Chawengkirttikul R. Ponpuak M. Charlermroj R. Boonyarattanakalin K. (2017). Synthesis and immunological studies of the lipomannan backbone glycans found on the surface of *Mycobacterium tuberculosis*. *J. Org. Chem.* 82 7190–7199. 10.1021/acs.joc.7b00703 28682637

[B50] Li Z. Feizi T. (2018). The neoglycolipid (NGL) technology-based microarrays and future prospects. *FEBS Lett.* 592 3976–3991. 10.1002/1873-3468.13217 30074246

[B51] Lisboa M. P. Khan N. Martin C. Xu F. F. Reppe K. Geissner A. (2017). Semisynthetic glycoconjugate vaccine candidate against *Streptococcus pneumoniae* serotype 5. *Proc. Natl. Acad. Sci. U.S.A.* 114 11063–11068. 10.1073/pnas.1706875114 28973947 PMC5651752

[B52] Liu X. Lei Z. Liu D. J. Wang Z. X. (2016). Development of a sandwiched microarray platform for studying the interactions of antibiotics with *Staphylococcus aureus*. *Anal. Chim. Acta* 917 93–100. 10.1016/j.aca.2016.02.038 27026605

[B53] Maolanon N. N. Blaise M. Sorensen K. K. Thygesen M. B. Cló E. Sullivan J. T. (2014). Lipochitin oligosaccharides immobilized through oximes in glycan microarrays bind LysM proteins. *Chembiochem* 15 425–434. 10.1002/cbic.201300520 24436194

[B54] Marchetti R. Malinovska L. Lameignere E. Adamova L. de Castro C. Cioci G. (2012). *Burkholderia cenocepacia* lectin A binding to heptoses from the bacterial lipopolysaccharide. *Glycobiology* 22 1387–1398. 10.1093/glycob/cws105 22763039

[B55] Marimon J. M. Monasterio A. Ercibengoa M. Pascual J. Prieto I. Simón L. (2010). Antibody microarray typing, a novel technique for *Streptococcus pneumoniae* serotyping. *J. Microbiol. Methods* 80 274–280. 10.1016/j.mimet.2010.01.011 20093147

[B56] Marotte K. Sabin C. Preville C. Moume-Pymbock M. Wimmerova M. Mitchell E. P. (2007). X-ray structures and thermodynamics of the interaction of PA-IIL from *Pseudomonas aeruginosa* with disaccharide derivatives. *ChemMedChem* 2 1328–1338. 10.1002/cmdc.200700100 17623286

[B57] Martin C. E. Broecker F. Eller S. Oberli M. A. Anish C. Pereira C. L. (2013a). Glycan arrays containing synthetic *Clostridium difficile* lipoteichoic acid oligomers as tools toward a carbohydrate vaccine. *Chem. Commun.* 49 7159–7161. 10.1039/c3cc43545h 23836132

[B58] Martin C. E. Broecker F. Oberli M. A. Komor J. Mattner J. Anish C. (2013b). Immunological evaluation of a synthetic *Clostridium difficile* oligosaccharide conjugate vaccine candidate and identification of a minimal epitope. *J. Am. Chem. Soc.* 135 9713–9722. 10.1021/ja401410y 23795894

[B59] Menova P. Sella M. Sellrie K. Pereira C. L. Seeberger P. H. (2018). Identification of the minimal glycotope of *Streptococcus pneumoniae* 7F capsular polysaccharide using synthetic oligosaccharides. *Chemistry* 24 4181–4187. 10.1002/chem.201705379 29333751

[B60] Mondani L. Delannoy S. Mathey R. Piat F. Mercey T. Slimani S. (2016). Fast detection of both O157 and non-O157 shiga-toxin producing *Escherichia coli* by real-time optical immunoassay. *Lett. Appl. Microbiol.* 62 39–46. 10.1111/lam.12503 26432989

[B61] Mondani L. Roupioz Y. Delannoy S. Fach P. Livache T. (2014). Simultaneous enrichment and optical detection of low levels of stressed *Escherichia coli* O157:H7 in food matrices. *J. Appl. Microbiol.* 117 537–546. 10.1111/jam.12522 24738929

[B62] Moonens K. Remaut H. (2017). Evolution and structural dynamics of bacterial glycan binding adhesins. *Curr. Opin. Struct. Biol.* 44 48–58. 10.1016/j.sbi.2016.12.003 28043017

[B63] Morley M. Molony C. M. Weber T. M. Devlin J. L. Ewens K. G. Spielman R. S. (2004). Genetic analysis of genome-wide variation in human gene expression. *Nature* 430 743–747. 10.1038/nature02797 15269782 PMC2966974

[B64] Neelamegham S. Aoki-Kinoshita K. Bolton E. Frank M. Lisacek F. Lutteke T. (2019). Updates to the symbol nomenclature for glycans guidelines. *Glycobiology* 29 620–624. 10.1093/glycob/cwz045 31184695 PMC7335484

[B65] Oberli M. A. Hecht M. L. Bindschadler P. Adibekian A. Adam T. Seeberger P. H. (2011). A possible oligosaccharide-conjugate vaccine candidate for *Clostridium difficile* is antigenic and immunogenic. *Chem. Biol.* 18 580–588. 10.1016/j.chembiol.2011.03.009 21609839

[B66] Oberli M. A. Tamborrini M. Tsai Y. H. Werz D. B. Horlacher T. Adibekian A. (2010). Molecular analysis of carbohydrate-antibody interactions: case study using a *Bacillus anthracis* tetrasaccharide. *J. Am. Chem. Soc.* 132 10239–10241. 10.1021/ja104027w 20614885

[B67] Palma A. S. Feizi T. Zhang Y. Stoll M. S. Lawson A. M. Díaz-Rodríguez E. (2006). Ligands for the beta-glucan receptor, Dectin-1, assigned using “designer” microarrays of oligosaccharide probes (neoglycolipids) generated from glucan polysaccharides. *J. Biol. Chem.* 281 5771–5779. 10.1074/jbc.M511461200 16371356

[B68] Palma A. S. Liu Y. Zhang H. Zhang Y. McCleary B. V. Yu G. (2015). Unravelling glucan recognition systems by glycome microarrays using the designer approach and mass spectrometry. *Mol. Cell Prot.* 14 974–988. 10.1074/mcp.M115.048272 25670804 PMC4390274

[B69] Parameswarappa S. G. Reppe K. Geissner A. Menova P. Govindan S. Calow A. D. J. (2016). A semi-synthetic oligosaccharide conjugate vaccine candidate confers protection against *Streptococcus pneumoniae* Serotype 3 Infection. *Cell Chem. Biol.* 23 1407–1416. 10.1016/j.chembiol.2016.09.016 27818299 PMC5234679

[B70] Parro V. de Diego-Castilla G. Moreno-Paz M. Blanco Y. Cruz-Gil P. Rodríguez-Manfredi J. A. (2011). A microbial oasis in the hypersaline Atacama subsurface discovered by a life detector chip: implications for the search for life on Mars. *Astrobiology* 11 969–996. 10.1089/ast.2011.0654 22149750 PMC3242637

[B71] Parthasarathy N. DeShazer D. England M. Waag D. M. (2006). Polysaccharide microarray technology for the detection of *Burkholderia pseudomallei* and *Burkholderia mallei* antibodies. *Diagn. Microbiol. Infect. Dis.* 56 329–332. 10.1016/j.diagmicrobio.2006.04.018 16765554 PMC7127370

[B72] Parthasarathy N. DeShazer D. Peacock S. J. Wuthiekanun V. England M. J. Norris S. L. (2008). Application of polysaccharide microarray technology for the serodiagnosis of *Burkholderia pseudomallei* infection (melioidosis) in humans. *J. Carbohydr. Chem.* 27 32–40. 10.1080/07328300802030761

[B73] Poole J. Day C. J. von Itzstein M. Paton J. C. Jennings M. P. (2018). Glycointeractions in bacterial pathogenesis. *Nat. Rev. Microbiol.* 16 440–452. 10.1038/s41579-018-0007-2 29674747

[B74] Prados-Rosales R. Carreño L. Cheng T. Blanc C. Weinrick B. Malek A. (2017). Enhanced control of *Mycobacterium tuberculosis* extrapulmonary dissemination in mice by an arabinomannan-protein conjugate vaccine. *PLoS Pathog.* 13:e1006250. 10.1371/journal.ppat.1006250 28278283 PMC5360349

[B75] Puente-Sánchez F. Moreno-Paz M. Rivas L. A. Cruz-Gil P. García-Villadangos M. Gómez M. J. (2014). Deep subsurface sulfate reduction and methanogenesis in the Iberian Pyrite Belt revealed through geochemistry and molecular biomarkers. *Geobiology* 12 34–47. 10.1111/gbi.12065 24237661

[B76] Reinhardt A. Wehle M. Geissner A. Crouch E. C. Kang Y. Yang Y. (2016). Structure binding relationship of human surfactant protein D and various lipopolysaccharide inner core structures. *J. Struct. Biol.* 195 387–395. 10.1016/j.jsb.2016.06.019 27350640

[B77] Reinhardt A. Yang Y. Claus H. Pereira C. L. Cox A. D. Vogel U. (2015). Antigenic potential of a highly conserved *Neisseria meningitidis* lipopolysaccharide inner core structure defined by chemical synthesis. *Chem. Biol.* 22 38–49. 10.1016/j.chembiol.2014.11.016 25601073

[B78] Rivas L. A. García-Villadangos M. Moreno-Paz M. Cruz-Gil P. Gómez-Elvira J. Parro V. (2008). A 200-antibody microarray biochip for environmental monitoring: searching for universal microbial biomarkers through immunoprofiling. *Anal. Chem.* 80 7970–7979. 10.1021/ac8008093 18837515

[B79] Salton M. R. J. Kim K. S. (1996). “Structure,” in *Medical Microbiology*, ed. Baron S., (Galveston, TX: University of Texas). 21413343

[B80] Saucedo N. M. Gao Y. N. Pham T. Mulchandani A. (2018). Lectin- and saccharide-functionalized nano-chemiresistor arrays for detection and identification of pathogenic bacteria infection. *Biosens. Basel* 8:63. 10.3390/bios8030063 29966294 PMC6165015

[B81] Seeberger P. H. Pereira C. L. Khan N. Xiao G. Diago-Navarro E. Reppe K. (2017). A semi-synthetic glycoconjugate vaccine candidate for carbapenem-resistant *Klebsiella pneumoniae*. *Angew Chem. Int. Engl.* 56 13973–13978. 10.1002/anie.201700964 28815890 PMC5819008

[B82] Semchenko E. A. Day C. J. Moutin M. Wilson J. C. Tiralongo J. Korolik V. (2012a). Structural heterogeneity of terminal glycans in *Campylobacter jejuni* lipooligosaccharides. *PloS one* 7:e40920. 10.1371/journal.pone.0040920 22815868 PMC3397941

[B83] Semchenko E. A. Moutin M. Korolik V. Tiralongo J. Day C. J. (2012b). Lectin array analysis of purified lipooligosaccharide: a method for the determination of molecular mimicry. *J. Glycomics Lipidomics* 2:103 10.4172/2153-0637.1000103

[B84] Solís D. Bovin N. V. Davis A. P. Jiménez-Barbero J. Romero A. Roy R. (2015). A guide into glycosciences: how chemistry, biochemistry and biology cooperate to crack the sugar code. *Biochim. Biophys. Acta Gen. Sub.* 1850 186–235. 10.1016/j.bbagen.2014.03.016 24685397

[B85] Sorensen K. K. Simonsen J. B. Maolanon N. N. Stougaard J. Jensen K. J. (2014). Chemically synthesized 58-mer LysM domain binds lipochitin oligosaccharide. *Chembiochem* 15 2097–2105. 10.1002/cbic.201402125 25154732

[B86] Stowell S. R. Arthur C. M. McBride R. Berger O. Razi N. Heimburg-Molinaro J. (2014). Microbial glycan microarrays define key features of host-microbial interactions. *Nat. Chem. Biol.* 10 470–476. 10.1038/nchembio.1525 24814672 PMC4158828

[B87] Sukhithasri V. Nisha N. Biswas L. Kumar V. A. Biswas R. (2013). Innate immune recognition of microbial cell wall components and microbial strategies to evade such recognitions. *Microbiol. Res.* 168 396–406. 10.1016/j.micres.2013.02.005 23578963

[B88] Sulak O. Cioci G. Lameignere E. Balloy V. Round A. Gutsche I. (2011). *Burkholderia cenocepacia* BC2L-C is a super lectin with dual specificity and proinflammatory activity. *PLoS Pathog.* 7:e1002238. 10.1371/journal.ppat.1002238 21909279 PMC3164656

[B89] Tamborrini M. Bauer M. Bolz M. Maho A. Oberli M. A. Werz D. B. (2011). Identification of an African *Bacillus anthracis* lineage that lacks expression of the spore surface-associated anthrose-containing oligosaccharide. *J. Bacteriol.* 193 3506–3511. 10.1128/JB.00078-11 21571994 PMC3133313

[B90] Tao S. C. Li Y. Zhou J. Qian J. Schnaar R. L. Zhang Y. (2008). Lectin microarrays identify cell-specific and functionally significant cell surface glycan markers. *Glycobiology* 18 761–769. 10.1093/glycob/cwn063 18625848 PMC2733773

[B91] Templier V. Livache T. Boisset S. Maurin M. Slimani S. Mathey R. (2017). Biochips for direct detection and identification of bacteria in blood culture-like conditions. *Sci. Rep.* 7:9457. 10.1038/s41598-017-10072-z 28842712 PMC5572712

[B92] Thirumalapura N. R. Morton R. J. Ramachandran A. Malayer J. R. (2005). Lipopolysaccharide microarrays for the detection of antibodies. *J. Immunol. Methods* 298 73–81. 10.1016/j.jim.2005.01.004 15847798

[B93] Thirumalapura N. R. Ramachandran A. Morton R. J. Malayer J. R. (2006). Bacterial cell microarrays for the detection and characterization of antibodies against surface antigens. *J. Immunol. Methods* 309 48–54. 10.1016/j.jim.2005.11.016 16423364

[B94] Tong M. Jacobi C. E. van de Rijke F. M. Kuijper S. van de Werken S. Lowary T. L. (2005). A multiplexed and miniaturized serological tuberculosis assay identifies antigens that discriminate maximally between TB and non-TB sera. *J. Immunol. Methods* 301 154–163. 10.1016/j.jim.2005.04.004 15979638

[B95] van der Es D. Berni F. Hogendorf W. F. J. Meeuwenoord N. Laverde D. van Diepen A. (2018). Streamlined synthesis and evaluation of teichoic acid fragments. *Chemistry* 24 4014–4018. 10.1002/chem.201800153 29389054 PMC5887911

[B96] Varki A. Cummings R. D. Aebi M. Packer N. H. Seeberger P. H. Esko J. D. (2015). Symbol Nomenclature for graphical representations of glycans. *Glycobiology* 25 1323–1324. 10.1093/glycob/cwv091 26543186 PMC4643639

[B97] Wang D. Carroll G. T. Turro N. J. Koberstein J. T. Kovac P. Saksena R. (2007). Photogenerated glycan arrays identify immunogenic sugar moieties of *Bacillus anthracis* exosporium. *Proteomics* 7 180–184. 10.1002/pmic.200600478 17205603

[B98] Wang D. Liu S. Trummer B. J. Deng C. Wang A. (2002). Carbohydrate microarrays for the recognition of cross-reactive molecular markers of microbes and host cells. *Nat. Biotechnol.* 20 275–281. 10.1038/nbt0302-275 11875429

[B99] Wang N. Hirata A. Nokihara K. Fukase K. Fujimoto Y. (2016). Peptidoglycan microarray as a novel tool to explore protein-ligand recognition. *Biopolymers* 106 422–429. 10.1002/bip.22807 26773558

[B100] Wesener D. A. Dugan A. Kiessling L. L. (2017). Recognition of microbial glycans by soluble human lectins. *Curr. Opin. Struct. Biol.* 44 168–178. 10.1016/j.sbi.2017.04.002 28482337 PMC6688470

[B101] Wesener D. A. Wangkanont K. McBride R. Song X. Kraft M. B. Hodges H. L. (2015). Recognition of microbial glycans by human intelectin-1. *Nat. Struct. Mol. Biol.* 22 603–610. 10.1038/nsmb.3053 26148048 PMC4526365

[B102] Wolter A. Niessner R. Seidel M. (2008). Detection of *Escherichia coli* O157:H7, *Salmonella typhimurium*, and *Legionella pneumophila* in water using a flow-through chemiluminescence microarray readout system. *Anal. Chem.* 80 5854–5863. 10.1021/ac800318b 18578502

[B103] Yasuda E. Tateno H. Hirabayashi J. Iino T. Sako T. (2011). Lectin microarray reveals binding profiles of *Lactobacillus casei* strains in a comprehensive analysis of bacterial cell wall polysaccharides. *Appl. Environ. Microbiol.* 77 4539–4546. 10.1128/AEM.00240-11 21602390 PMC3127709

[B104] Zhang H. Palma A. S. Zhang Y. Childs R. A. Liu Y. Mitchell D. A. (2016). Generation and characterization of beta1,2-gluco-oligosaccharide probes from *Brucella abortus* cyclic beta-glucan and their recognition by C-type lectins of the immune system. *Glycobiology* 26 1086–1096. 10.1093/glycob/cww041 27053576 PMC5072146

[B105] Zheng L. B. Wan Y. Qi P. Sun Y. Zhang D. Yu L. M. (2017). Lectin functionalized ZnO nanoarrays as a 3D nano-biointerface for bacterial detection. *Talanta* 167 600–606. 10.1016/j.talanta.2017.03.007 28340767

[B106] Zheng R. B. Jégouzo S. A. F. Joe M. Bai Y. Tran H. A. Shen K. (2017). Insights into interactions of mycobacteria with the host innate immune system from a novel array of synthetic mycobacterial glycans. *ACS Chem. Biol.* 12 2990–3002. 10.1021/acschembio.7b00797 29048873 PMC5735379

[B107] Zhu H. Bilgin M. Bangham R. Hall D. Casamayor A. Bertone P. (2001). Global analysis of protein activities using proteome chips. *Science* 293 2101–2105. 10.1126/science.1062191 11474067

